# Efficient Microwave‐Assisted Hydrolytic Recycling of Poly(L‐Lactic Acid)

**DOI:** 10.1002/cssc.202502366

**Published:** 2026-02-26

**Authors:** Federica Santulli, Maëlie Chauvin, Rosaria Schettini, Marina Lamberti, Frédéric de Montigny, Christophe M. Thomas, Mina Mazzeo

**Affiliations:** ^1^ Department of Chemistry and Biology “A. Zambelli” University of Salerno Fisciano (Salerno) Italy; ^2^ Chimie ParisTech CNRS PSL University Institut de Recherche de Chimie Paris Paris France

**Keywords:** chemical recycling, circular economy, hydrolysis, poly(L‐lactic acid), polyesters

## Abstract

Poly(L‐Lactic acid) (PLLA) is a bio‐based and biodegradable thermoplastic polymer widely recognized as a leading sustainable alternative to conventional petroleum‐based plastics. While its environmental benefits are well established, PLLA faces challenges in end‐of‐life management due to its slow degradation in natural conditions and the harsh requirements of industrial composting. This study introduces an efficient chemical recycling strategy for PLLA based on hydrolysis reactions performed both in solution and under solvent‐free conditions, catalyzed by a homoleptic phenoxy‐imine pyridine zinc complex. Both conventional and microwave‐assisted heating methods were evaluated. Hydrolysis in solution exhibited consistent degradation rates across various solvents, irrespective of the heating technique. In contrast, microwave‐assisted heterogeneous hydrolysis significantly improved both reaction rate and selectivity. Notably, this process enables the direct conversion of postconsumer PLLA products into lactic acid under mild reaction conditions, without the need for additional solvents or pressure build‐up. The catalytic approach demonstrates a scalable, energy‐efficient pathway for closing the PLLA lifecycle, offering a viable solution for industrial monomer recovery with low waste generation.

## Graphical abstract

Microwave‐assisted hydrolysis of poly(lactic acid) promoted by a zinc catalyst. A highly efficient procedure performed under mild reaction conditions and in the absence of solvents aligning with green chemistry principles.

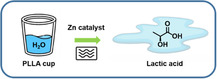

## 1 Introduction

The escalating accumulation of plastic solid waste in the environment, primarily driven by the inherent durability and resistance to degradation of conventional polymers, has become a pressing global concern [1, 2, 3]. Despite the well‐documented environmental and economic advantages of plastic recycling, effective end‐of‐life management strategies remain insufficient. Thus, the great majority of plastic waste continues to be landfilled, incinerated, or inadvertently released into the natural ecosystems. Only a small fraction is currently recycled, mostly through mechanical processes that often lead to downcycling, with a significant loss in material performance [4, 5].

Over the past two decades, research has increasingly redefined plastic waste as a valuable resource, catalyzing innovation in recycling technologies [6, 7] and promoting the development of biodegradable and/or chemically recyclable polymer [8, 9, 10, 11, 12, 13]. Among these, poly(L‐lactic acid) (PLLA) has emerged as a prominent bio‐based aliphatic polyester, offering a unique combination of biodegradability, biocompatibility, and favorable mechanical properties. The combination of those attributes has positioned PLLA as a leading sustainable alternative to petroleum‐based plastics, with applications across packaging, textiles, agriculture, and biomedical devices [14]. More than 670,000 tons of PLLA are produced worldwide every year, representing 37% of the total bio‐based plastic produced [15, 16]. However, the rapid growth in demand and production has raised concerns regarding PLLA's long‐term environmental impact and the urgent need for efficient end‐of‐life management strategies [17, 18]. Its limited thermal and hydrolytic stability poses challenges for mechanical recycling [19], and although industrial composting is technically feasible, life cycle assessment (LCA) studies consistently indicate that chemical recycling offers superior environmental outcomes [20, 21]. Moreover, PLLA production is based on fermentable sugars obtained from food crops, thus directly competing with agricultural feedstocks and raising legitimate concerns about food security. These limitations underline the critical need for scalable, energy‐efficient recycling technologies that can close the material loop and reduce reliance on virgin feedstocks, boosting the sustainability of PLLA in its lifecycle.

Chemical recycling of PLLA into low‐molecular‐weight molecules has emerged as a promising strategy to retain the intrinsic value of the bioderived feedstocks [22, 23, 24]. Several depolymerization pathways have been employed, including hydrolysis [23, 24, 25], alcoholysis [26, 27, 28, 29, 30, 31, 32, 33, 34], thermal degradation [35], chemical recycling to monomer (CRM) [36, 37], and enzymatic processes [38]. Among these, hydrolysis to lactic acid is especially appealing due to its inherent circularity. Lactic acid serves as the primary precursor of lactide, which is the monomer industrially used for PLLA synthesis. Moreover, the recovery of lactic acid via chemical recycling is more cost‐effective than its production by starch fermentation, which accounts for approximately 50% of the total production cost of the monomer [23, 39]. Furthermore, lactic acid is recognized as a key platform molecule in bioeconomy, serving as a precursor for a wide range of value‐added chemicals, including acrylic acid, pyruvic acid, and alkyl lactates [40].

However, under neutral conditions, PLLA hydrolysis typically requires harsh reaction conditions (temperature exceeding 180°C, pressures above 1.5 MPa, and large water excesses). This results in an energy‐intensive process and increases the risk of racemization or degradation of the recovered monomer [41].

Catalytic hydrolysis of PLLA under milder conditions has been explored using acidic or alkaline systems. For example, complete depolymerization can be achieved with 0.6 M NaOH at 160°C within 30 min [42], whereas milder thermal conditions using 0.3 M Ca(OH)_2_ results in only 8.3% degradation after 12 h at 90°C [43]. Acidic media, such as sulfuric acid, assure comparable reaction kinetics but introduce significant drawbacks, such as corrosion risks and the need for postreaction neutralization and separation steps. More recently, ionic liquids (e.g., 1‐butyl‐3‐methylimidazolium acetate [Bmim][OAc]) have shown high hydrolytic efficiency, achieving 94% degradation within 2 h at 130°C. However, the high catalyst loading (up to 50 wt% relative to PLLA) and associated costs limit industrial scalability [44].

Interestingly, although metal‐based catalysts are widely employed in PLLA alcoholysis [45, 46, 47, 48, 49], their application in hydrolytic depolymerization remains underexplored, despite their potential for high activity under mild conditions, along with the advantages of recovery and reuse. Heterogeneous ZnO could be considered an exception, as it demonstrated promising performance by achieving 93% PLLA degradation at 130°C in 24 h using a large excess of water (PLLA:H_2_O = 1:500 w/w). This result highlights the potential of metal‐based systems in hydrolytic recycling [50].

In parallel, microwave‐assisted depolymerization has emerged as a powerful technique to accelerate polymer degradation [51, 52, 53]. For example, PLLA alcoholysis with diols under microwave irradiation has shown higher performance compared to traditional thermal methods, offering faster reaction kinetics and improved energy efficiency [54, 55, 56]. Similarly, microwave‐assisted alkaline hydrolysis has achieved high depolymerization efficiency under relatively mild conditions using phase‐transfer catalysts [57]. These effects are due to ionic drift mechanisms, whereby microwave fields increase the mobility of polar species, favoring nucleophilic attack on PLLA ester bonds and thus accelerating alcoholysis or hydrolysis [58]. These findings underscore the significant potential of integrating metal‐based catalysis with microwave‐assisted techniques to develop scalable and energy‐efficient recycling processes for PLLA.

In this work, we report the first application of a zinc‐based metal complex for the hydrolysis of PLLA under microwave‐assisted conditions. This strategy enables efficient recovery of lactic acid with minimal generation of waste, supports catalyst recyclability, and offers a scalable, environmentally responsible solution for end‐of‐life PLLA management.

## 2 Results and Discussion

### 2.1 Hydrolysis of PLLA in Solution

**SCHEME 1:**
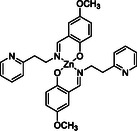
Structure of the zinc complex **1** used for this study.

For the catalytic hydrolysis of PLLA, we selected the homoleptic zinc complex **1** featuring a phenoxy–imino–pyridine ligand (Scheme 1, Figures S1–S6). This complex has previously demonstrated high thermal stability and resistance to protic media in PLLA alcoholysis reactions, making it a promising candidate for hydrolytic applications [45, 55]. To evaluate its suitability under aqueous conditions, we first assessed its stability using ^1^H NMR spectroscopy. Upon dissolution in DMSO‐d_6_ followed by the addition of H_2_O (30 equiv.), no changes were observed in proton resonances of the complex (Figure S7), indicating its robustness under hydrolytic conditions. These properties underscore the potential of complex **1** as an effective catalyst for PLLA depolymerization.

Commercial‐grade PLLA, obtained from postconsumer transparent cups, was selected as the substrate for this study. Its molecular characteristics were determined by gel permeation chromatography (GPC), yielding a number‐average molecular weight (*M*_*n*_) of 51 kDa and a dispersity (*Ð*) of 1.9 (Figure S9). Thermal analysis (Figures S10–S11) revealed a glass transition temperature (*T*_g_) of 59°C and a melting temperature (*T*_m_) of 149°C, consistent with typical properties of semicrystalline PLLA.

**FIGURE 1:**
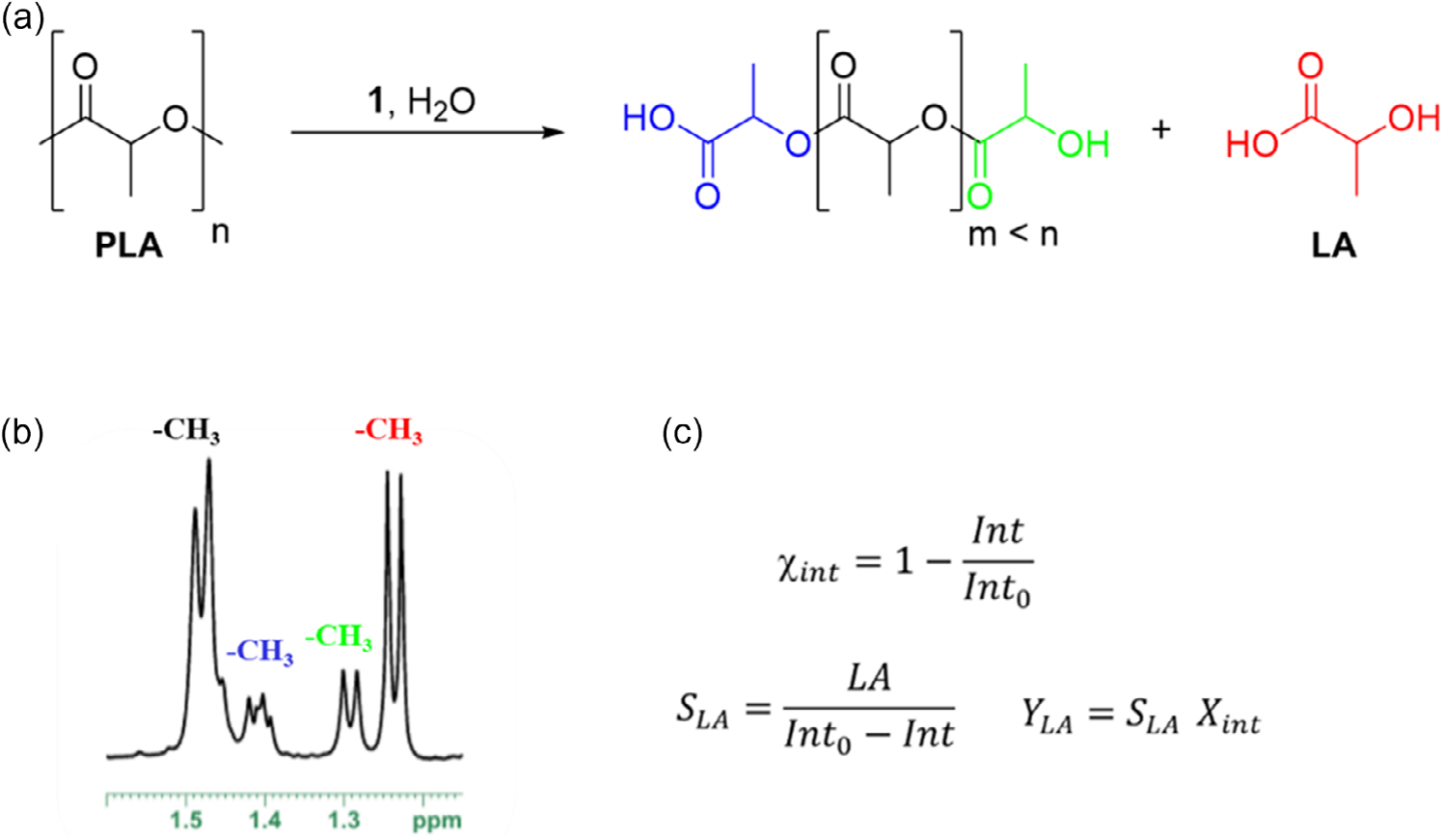
Hydrolysis of PLLA (a), ^1^H NMR spectrum (400 MHz, 298 K, DMSO‐d_6_) of mixture of reaction (b), equations to calculate the conversion of internal methine units (X_int_), lactic acid selectivity (S_LA_), and lactic acid yield (Y_LA_). A note has been added to clarify the meaning of the parameters used [59] (c).

Generally, PLLA hydrolysis proceeds via nucleophilic attack of water on the ester bonds along the polymer backbone, leading to chain scission and the formation of lactic acid and lower molecular weight oligomers (Figure 1a).

Hydrolysis reactions were conducted using commercially available PLLA, that had been cut into flakes measuring approximately 0.5 cm^2^. For each reaction, PLLA was dissolved in 3.1 ml of technical‐grade, unpurified solvent, followed by the addition of 0.6 mol% of the complex **1** related to ester unit (L_2_Zn:PLLA = 0.04:1 w/w).

Water was added at a ratio of 5 equivalents per ester unit (H_2_O:PLLA = 1.2:1 w/w). The reaction mixture was then subjected to microwave irradiation at 150°C.

The progression of the hydrolysis reaction was monitored using ^1^H NMR spectroscopy. Across all experiments, the reaction mixture comprised lactic acid, lactic acid oligomers, and residual undegraded PLLA, with no unsaturated byproducts (Figure S12). The signals characteristics of all these species are clearly distinguished in the methyl region (1.1–1.6 ppm), which shows diagnostic resonances attributable to internal PLLA units, chain‐ends of oligomers, and free lactic acid (Figure 1b). Quantitative analysis of polymer degradation (X_int_), lactic acid yield (Y_LA_), and selectivity (S_LA_) was performed following established protocols previously developed for alcoholysis reactions (Figure 1c and Figure S12) [27, 30, 60]. The reliability of the NMR measurements was assessed by repeating selected experiments in the presence of tetrakis(trimethylsilyl)silane (TMSS) as an internal standard (Figure S13) [55] and by performing quantitative reverse HPLC analysis (Figures S14,S15, Table S1) [50, 61]. The results obtained were consistent with those from the initial analyses, confirming the accuracy of the method [55].

Solvent selection for microwave‐assisted processes was guided by two key parameters: the dielectric constant (*ε*), which reflects the ability of a solvent to store electrical energy, and the dielectric loss tangent (tan *δ*), which measures its capacity to convert microwave energy into heat [62, 63]. Based on these criteria, *N*,*N*‐dimethylformamide (DMF) and dimethyl sulfoxide (DMSO), two polar aprotic solvents with strong microwave absorption capabilities (tan *δ* = 0.161 for DMF; tan *δ* = 0.825 for DMSO), were selected [63, 64]. Both solvents ensured complete dissolution of the PLLA substrate, facilitating uniform reaction conditions.

In DMF, PLLA degradation reached 94% within 2 h, with a lactic acid yield of 72% (entry 1, Table 1). In contrast, the absence of catalyst resulted in minimal conversion (X_int_ = 7%), confirming the essential role of complex **1** in promoting hydrolysis (entry 2, Table 1).

**TABLE 1:** Hydrolysis of PLLA with **1** in different solvents.^a^

Entry	Solvent	P_in_ b,W	ΔP^b^,atm	X_int_ c, %	S_LA_ c, %	Y_LA_ c, %
1	DMF	30	0	94	76	72
2^d^	DMF	30	0	7	9	3
3	DMSO	30	0	47	42	20
4	1,3‐dioxolane	126	6	40	40	16
5	Me‐THF	100	4	55	46	34
6	Anisole	40	0	12	13	2
7^e^	DMF	—	—	94	79	74
8^e^	DMSO	—	—	44	42	20

a All reactions were carried out in air by using 10 μmol of **1** (0.6 mol % relative to ester linkages) 0.116 g of PLLA from transparent cup, 0.14 mL of H_2_O (5 equiv. with respect to the lactyl units) in 3.1 mL of solvent ([lactyl unit] = 0.5 M) in a microwave reactor at 150°C for 2 h.

b Microwave input power (P_in_) and pressure variation (ΔP) were monitored in real time by the CEM Discover 2.0 system. MW corresponds to the value required to reach and maintain the indicated reaction temperature.

c Determined by ^1^H NMR spectroscopy using the equations in Figure 1.

d Blank experiment with no catalyst.

e Conventional heating with bath oil.

In DMSO, moderate catalytic activity was observed (entry 3, Table 1), with 47% PLLA degradation and a lactic acid yield of 20% after 2 h. The reduced efficiency may be attributed to strong solvent coordination to the zinc center, which could limit catalyst availability, or to the relatively high viscosity of DMSO, potentially hindering the diffusion of water and catalyst throughout the polymer matrix. Reactions in DMF and DMSO required relatively low microwave power inputs (˜30 W) to maintain 150°C, and no pressure build‐up was detected. This is attributed to their high boiling points (153°C for DMF and 189°C for DMSO), which minimize solvent vaporization at these temperatures.

These results prompted the evaluation of more sustainable solvents, such as 1,3‐dioxolane and 2‐methyltetrahydrofuran (Me‐THF). Both solvents successfully solubilized PLLA and yielded degradation efficiencies comparable to DMSO (40%–55%, entries 4, 5, Table 1). However, due to their lower polarity, higher microwave power inputs (>100 W) were required to achieve and maintain the target temperature.

Moreover, due to their relatively low boiling points (74°C for 1,3‐dioxolane and 78°C for Me‐THF), substantial pressure build‐up was observed, with values reaching up to 6 atm [65]. Anisole was also tested, but its immiscibility with water and limited ability to dissolve PLLA resulted in low reactivity (X_int_ = 12%, entry 6).

To assess the specific contribution of microwave irradiation, key experiments were repeated under conventional heating by using an oil bath (entries 7 and 8, Table 1). In both DMF and DMSO, no significant differences in yield were observed, suggesting that when PLLA is fully solubilized, water–polymer interactions are maximized, and the benefits of microwave heating (MW)are less pronounced under these conditions. Nonetheless, the presence of the catalyst remained crucial for effective hydrolysis under all conditions tested.

To identify low‐toxicity and cost‐effective solvent alternatives, acetone was selected due to its favorable safety profile, availability and compatibility with industrial processes. Piemonte and Gironi previously reported that acetone exhibits superior PLLA solubilization in the presence of a large amount of water compared to ethyl acetate [66]. Under initial reaction conditions (0.5 M PLLA, 2 h, 120°C), only limited polymer degradation was observed (21%, entry 1, Table 2). This result can be attributed to acetone's poor microwave absorption capacity, as indicated by its low dielectric loss tangent (tan *δ* = 0.054) [63], which required a high microwave power input (120 W) to maintain the target temperature. Moreover, due to its low boiling point (56°C), the reaction system reached internal pressures up to 6 atm under microwave irradiation.

**TABLE 2:** Hydrolysis of PLLA with **1** in acetone.^a^

Entry	Concentration, M	Acetone:H_2_O, v/v	P_in_ b, W	ΔP^b^, atm	Time, h	X_int_ c, %	S_LA_ c, %	Y_LA_ c, %
1	0.5	21:1	120	6	2	21	25	5
2	1.5	7:1	40	4	2	41	49	20
3	3.0	3.5:1	20	0	2	49	60	29
4	3.0	3.5:1	20	0	5	86	74	64
5^d^	3.0	3.5:1	20	0	16	98	92	90
6^e^	3.0	3.5:1	20	0	2	40	46	19
7^f^	3.0	3.5:1	20	0	2	39	45	18

a All reactions were carried out in air by using 10 μmol of **1** (0.6 mol % relative to ester linkages) 0.116 g of PLLA from transparent cup, 0.14 mL of H_2_O (5 equiv. with respect to the lactyl units) in acetone (3.1 mL for [lactyl unit] = 0.5 M, 1.0 mL for [lactyl unit] = 1.5 M, and 0.5 mL for [lactyl unit] = 3.0 M) using microwave reactor at 120°C.

b Microwave input power (P_in_) and pressure variation (ΔP) were monitored in real time by the CEM Discover 2.0 system. MW corresponds to the values required to reach and maintain the indicated reaction temperature.

c Determined by ^1^H NMR spectroscopy using the equations in Figure 1.

d Reaction time not optimized.

e ZnO was used instead of **1**.

f Zn(OAc)_2_ was used instead of **1**.

To overcome these limitations, the volume of acetone was reduced to the minimum required to ensure complete solubilization of PLLA at the target temperature, while maintaining a constant PLLA:H_2_O ratio. This modification led to a threefold increase in polymer concentration (3.0 M, entry 3, Table 2) and a corresponding increase in the relative proportion of water within the reaction medium. The higher water content, combined with its superior dielectric loss (tan δ = 0.123) [63, 64] compared to acetone, significantly improved microwave energy absorption. As a result, the target reaction temperature was achieved and maintained with a significantly lower microwave power input of only 20 W.

**FIGURE 2:**
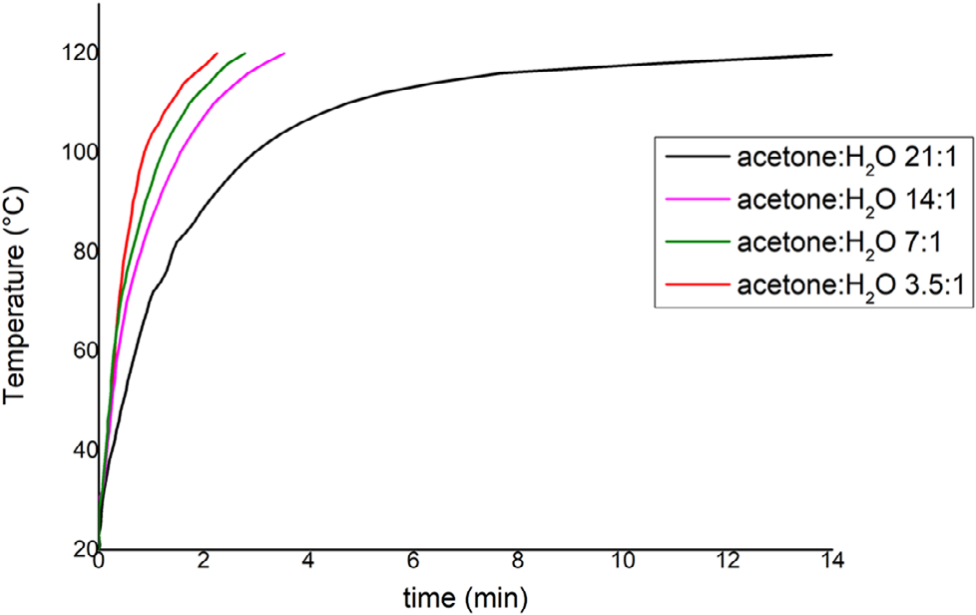
Temperatures profiles of solutions with different acetone:H_2_O volume ratios during microwave irradiation. The maximum microwave input power was set to 150 W.

The different responses of acetone and H_2_O to microwave irradiation are illustrated in Figure 2, where the temperature profiles of both solvents under microwave irradiation were monitored over time. Pure acetone required 14 min to reach 120°C, whereas H_2_O achieved the same temperature in just 2 min. As the amount of acetone decreased and the ratio H_2_O: acetone increased, the heating rate improved significantly. This behavior reflects enhanced dielectric properties of the solvent mixture, which become more favorable for microwave absorption with increasing H_2_O content (Figure 2).

Moreover, the higher PLLA concentration promoted stronger interactions between PLLA chains, H_2_O, and the catalyst, thereby improving overall depolymerization efficiency. Notably, no pressure build‐up was observed during microwave irradiation under these conditions.

Further optimization by extending the reaction time to 16 h (entry 5, Table 2) led to near‐complete depolymerization (98%) and high yield of lactic acid (90%), demonstrating the robustness and efficiency of the catalytic system. Although direct comparisons with literature data are complicated by variations in water and/or catalyst loadings, the catalytic performance of complex **1** was further benchmarked against two representative systems, ZnO and Zn(OAc)_2_, previously employed for PLLA degradation [50]. Under our standardized conditions, both systems showed significantly lower PLLA degradation (X_int_ ≈ 40%) compared to complex **1** (compare entry 3 with entries 6 and 7 in Table 2), confirming that complex **1** ranks among the most efficient catalysts reported to date under sustainable relevant conditions.

**FIGURE 3:**
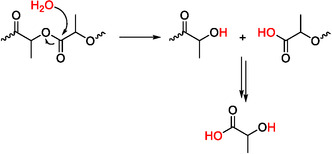
Possible degradation mechanism of PLLA via bulk erosion, involving the formation of oligomeric species followed by lactic acid formation.

To gain mechanistic insight into the hydrolytic degradation of PLLA in solution, the molecular weights of the degradation products were analyzed via GPC. Across all tested conditions, regardless of the extent of polymer degradation (entries 1–4, Table 2), the resulting species consistently exhibited molecular weights below 1.0 kDa (Table S2). This observation suggests that hydrolysis proceeds through a homogeneous erosion mechanism, wherein polymer chains are uniformly cleaved in solution, rather than through surface erosion (Figure 3).

Such behavior is consistent with previous findings by Liu et al., who reported similar degradation profiles for PLLA solubilized in dioxane [43].

### 2.2 Hydrolysis of PLLA under Solvent‐Free Conditions

**FIGURE 4:**
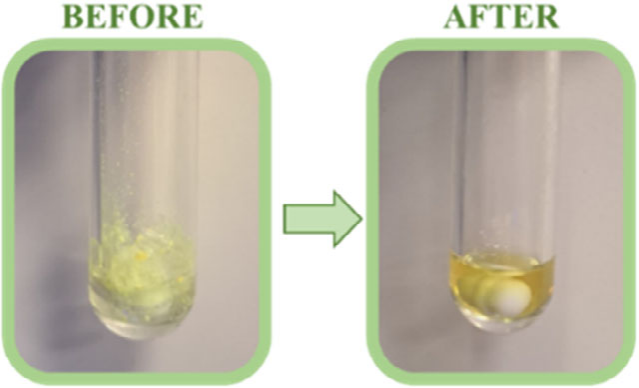
Images captured before and after the hydrolysis reaction conducted under solvent‐free conditions.

To further improve the sustainability of the recycling process, we investigated the hydrolysis of PLLA under solvent‐free conditions (Figure 4, Table 3), using a molar ratio of PLLA:L_2_Zn:H_2_O of 1:0.006:5 (corresponding to a w/w ratio of 1:0.04:1.2). These reaction conditions are significantly more demanding than those commonly reported in literature, which often rely on large excesses of water, elevated temperatures, and high pressure to facilitate hydrolysis [23, 24].

**TABLE 3:** Hydrolysis of PLLA with **1** under solvent‐free conditions.^a^

Entry	H_2_O, equiv.	Temperature, °C	P_in_ b, W	Time, h	X_int_ c, %	S_LA_ c, %	Y_LA_ c, %
1	5	130	30	4	92	57	52
2^d^	5	130	50	4	62	46	29
3^e^	5	130	—	4	76	62	47
4d,, e	5	130	—	4	—	—	—
5	15	130	30	4	98	94	93
6	5	150	20	2	100	85	85
7	10	150	25	2	100	92	92
8	15	150	25	2	100	98	98

a All reactions were carried out in air by using 10 μmol of **1** (0.6 mol % relative to ester linkages) 0.116 g of PLLA from transparent cup, 0.14 mL of H_2_O (5 equiv. with respect to the lactyl units) or 0.42 mL of H_2_O (15 equiv. with respect to the lactyl units) under solvent‐free conditions using microwave reactor.

b Microwave input power (P_in_) and pressure variation (ΔP) were monitored in real time by the CEM Discover 2.0 system. MW corresponds to the values required to reach and maintain the indicated reaction temperature.

c Determined by ^1^H NMR spectroscopy using the equations in Figure 1.

d Blank experiment with no catalyst.

e Traditional heating with bath oil.

Under microwave‐assisted heating at 130°C for 4 h (entry 1, Table 3), near‐complete degradation of PLLA was achieved (X_int_ = 92%) with a lactic acid yield of 52%. In the absence of the zinc catalyst, degradation efficiency dropped to 62% (entry 2, Table 3) and a yield of lactic acid of 29%, confirming the beneficial role of the catalyst in enhancing both activity and selectivity. Interestingly, when conventional heating was used instead of microwave irradiation (entry 3, Table 3), the degradation efficiency decreased (X_int_ = 76%), while the product selectivity remained comparable to that observed under microwave‐assisted conditions.

For comparison, Barbaro et al. reported the hydrolysis of PLLA using a heterogeneous ZnO catalyst at the same temperature under conventional heating. However, it required 24 h to reach 93% degradation, despite using a lactyl unit:ZnO molar ratio of 1:0.9, approximately 150 times higher than the catalyst amount employed in our study [50]. Finally, when both the catalyst and microwave heating were omitted (entry 4, Table 3), no degradation was observed. These data highlight the beneficial effect of combining the catalyst with microwave irradiation.

Given the critical importance of maintaining the optical purity of lactic acid obtained from hydrolysis, we evaluated the degree of racemization under the experimental conditions used in this study. Following solvent‐free, microwave‐assisted hydrolysis at 130°C (entry 1, Table 3), the recovered lactic acid was converted to its benzyl ester to facilitate precise chiral HPLC analysis (Figures S16, S17) [67]. The results revealed an enantiomeric excess of 98% of L‐lactic acid (Figure S18). This slight deviation from complete enantiopurity likely arises from the intrinsic stereochemical heterogeneity of the starting PLLA, rather than racemization during hydrolysis.

Supporting this view, homodecoupled ^1^H NMR analysis of the PLLA showed a *meso*‐dyad probability (P_*m*_) of 0.98 (Figure S8) [68], consistent with the presence of a minor fraction of stereodefects arising from *meso*‐lactide incorporation in the feedstock [69]. Overall, these results indicate that stereochemical integrity is largely preserved during hydrolysis and that the depolymerization conditions employed do not induce measurable racemization. This conclusion aligns with previous reports which show that significant racemization occurs only under markedly harsher conditions, such as elevated temperatures (250°C–350°C) or high‐pressure aqueous hydrolysis, whereas milder or microwave‐assisted protocols effectively preserve stereochemical integrity [57, 70].

To optimize the process at 130°C, the reaction was carried out with an increased amount of water (15 equiv. instead of 5 equiv.), resulting in a significant enhancement in lactic acid yield, which reached 93% (see entries 1 and 5, Table 3). Further elevation of the temperature to 150°C (entry 6, Table 3) enabled complete PLLA degradation (X_int_ = 100%) within 2 h, affording a lactic acid yield of 85%. The final reaction mixture consisted exclusively of lactic acid and its dimer (dilactic acid) in an 85:15 molar ratio (Figures S20, S21), with no detectable oligomeric species. The products obtained were efficiently purified by vacuum distillation to remove the residual catalyst (Figure S22), allowing recovery of approximately 70% of the reaction mixture, which was slightly enriched in lactic acid (95%). Notably, no evidence of lactic acid repolymerization was observed under these conditions, as confirmed by NMR analysis (Figure S23).

Increasing the water equivalents to 10 and 15 (entries 7 and P8, Table 3) led to quantitative production of lactic acid, demonstrating the scalability and efficiency of this solvent‐free, microwave‐assisted catalytic system.

**FIGURE 5:**
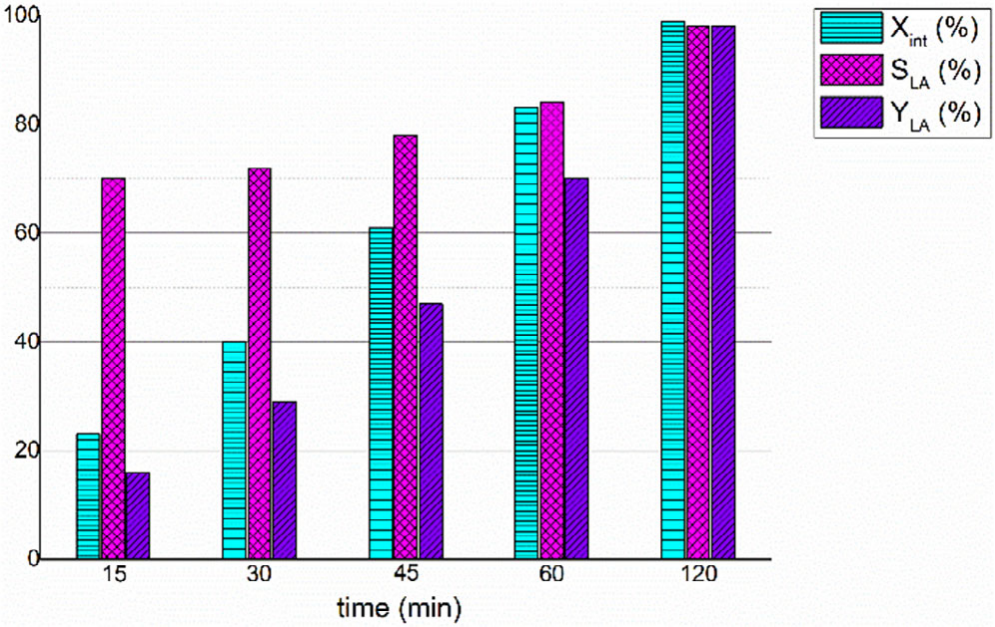
Time‐dependent trends of PLLA conversion (X_int_), lactic acid selectivity (S_LA_), and lactic acid yield (Y_LA_) during hydrolysis reactions of PLLA. Reaction conditions: 10 μmol of **1** (0.6 mol% relative to ester linkages), 0.116 g of PLLA from transparent cup, 0.42 mL of H_2_O (15 equiv. with respect to the lactyl units) using microwave heating (MW).

To elucidate the reaction mechanism of PLLA hydrolysis under solvent‐free conditions, a series of time‐dependent experiments were conducted at 150°C using 15 equiv. of water (Figure 5 and Table S3).

Notably, no pressure build‐up was observed during microwave irradiation for these reactions. At each time point, the reaction mixture was fully dissolved in DMSO, and the polymer degradation (X_int_), selectivity (S_LA_), and lactic acid yield (Y_LA_) were quantified by ^1^H NMR spectroscopy. As expected, both the degree of degradation and the lactic acid yield increased progressively with reaction time. For each reaction, the number‐average molecular weight of the residual polymer was analyzed by GPC. After 15 min, 23% of the polymer was degraded, and the *M*_*n*_ of the residual polymer dropped dramatically of 97% (*M*_n,GPC_ = 1.5 kDa), suggesting a bulk erosion mechanism, as observed for the hydrolysis reaction in solution (Figure 3).

**FIGURE 6:**
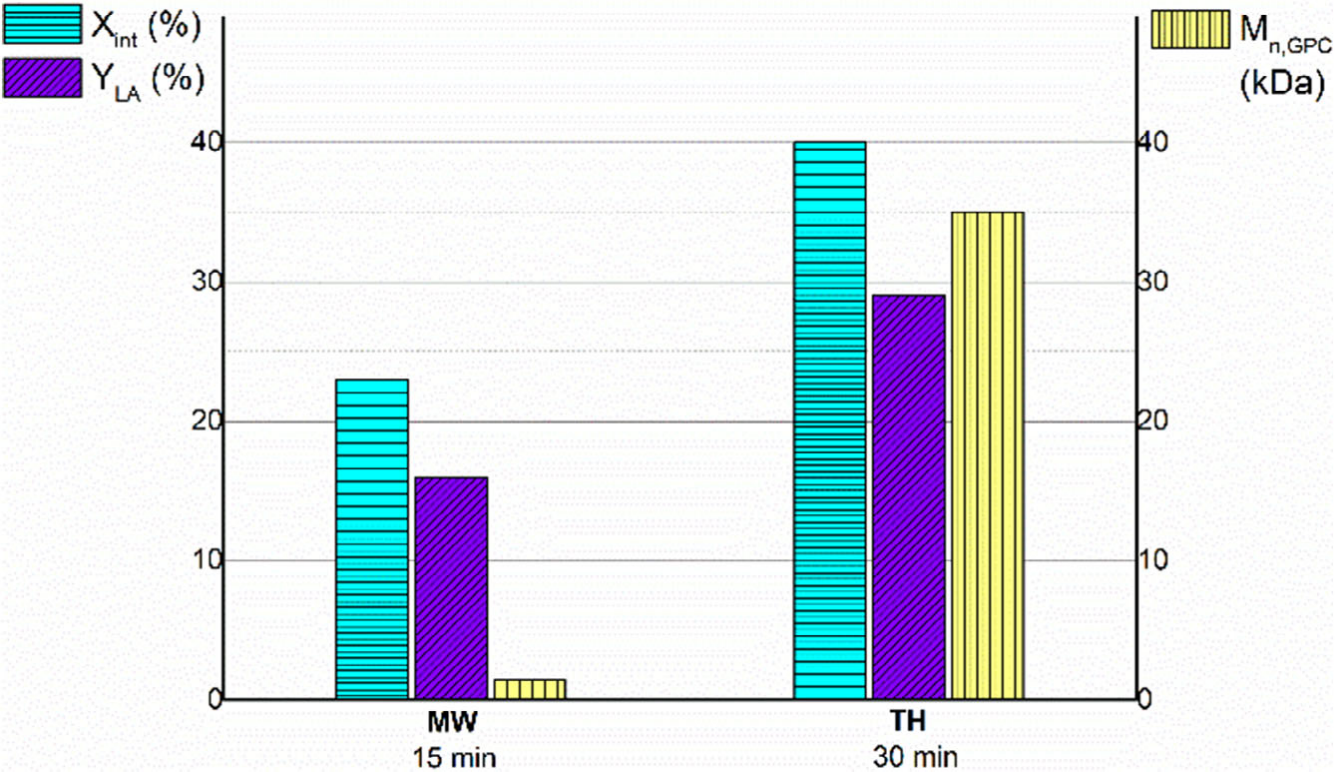
Time‐dependent trends of PLLA conversion (X_int_), lactic acid yield (Y_LA_), and molecular weight of the residual polymer (*M*_n__,__GPC_) during hydrolysis reaction of PLLA using microwave heating (MW) and traditional heating (TH).

In contrast, under conventional heating, the same degree of degradation was only achieved after 30 min, and the residual polymer retained a significantly higher molecular weight (*M*_n,GPC_ = 35 kDa), representing only a 30% reduction from the initial value (Figure 6 and Figure S24). This behavior is consistent with a surface erosion mechanism, also reported by Liu et al., who proposed that degradation primarily occurs at the polymer–water interface [43].

This different behavior observed under microwave heating compared to conventional heating may be explained by the heterogeneous nature of the reaction system under solvent‐free conditions. In such systems, microwave energy is absorbed selectively by components with high dielectric loss, such as water and lactic acid, while the solid polymer matrix absorbs energy less efficiently [57, 62]. This differential absorption can result in the formation of localized thermal gradients, so‐called hot‐spots, within the reaction medium [71, 72]. These hot‐spots facilitate deeper penetration of water molecules into the bulk polymer, promoting internal chain scission and enabling bulk erosion, rather than surface‐limited degradation (Figure 3).

Finally, the recyclability of the catalyst was evaluated under solvent‐free conditions. Hydrolysis was conducted at 130°C under microwave heating with 5 equivalents of water, following the protocol described for entry 1 in Table 3. The initial degradation step proceeded as previously outlined, achieving 92% PLLA conversion after 4 h. At this stage, an additional, equivalent amount of PLLA and water was introduced into the reaction mixture, resulting in a further conversion of 90% after 4 h. A third addition of PLLA and water afforded 88% conversion with a selectivity of 70%, thus demonstrating that the catalyst can be reused with only a moderate decline in activity and selectivity.

These solvent‐free conditions represent a highly promising approach for the chemical recycling of PLLA, offering significant advantages in terms of sustainability and process simplicity. By eliminating the need for organic solvents, minimizing reagent consumption and streamlining product recovery, all while avoiding pressure build‐up during the reaction, this strategy aligns closely with the principles of green chemistry. The ability to achieve quantitative depolymerization under mild conditions, with low catalyst loading and minimal water input, marks a substantial advancement toward scalable, environmentally friendly recycling technologies for bio‐based polymers.

## 3 Conclusion

In this study, we report the first application of a homoleptic zinc complex for the efficient hydrolysis of postconsumer PLLA into lactic acid. The catalyst enables near‐complete depolymerization under mild conditions, both in solution and under solvent‐free setups.

The choice of solvents strongly influences reaction efficiency: polar aprotic solvents such as DMF and acetone significantly enhance PLLA solubilization and water diffusion, thus promoting hydrolysis to low‐molecular weight oligomers and their subsequent conversion into lactic acid. Under these conditions, microwave irradiation showed limited impact compared to conventional heating. However, under solvent‐free conditions, microwave‐assisted heating markedly improved both reaction rate and selectivity, promoting bulk erosion through localized thermal gradients.

The most sustainable approach was achieved under solvent‐free conditions with minimal water input, yielding complete PLLA degradation in just 2 h at 150°C without pressure build‐up. This catalytic microwave‐assisted process offers several advantages compared to conventional chemical recycling techniques, including low catalyst loading, minimal water use, elimination of corrosive acids or bases, and simplified product recovery. These features position the method as a promising strategy for the chemical recycling of PLLA.

## 4 Experimental Section

### 4.1 Hydrolysis of PLLA in Solution

The hydrolysis of PLLA was carried out in a microwave reactor (CEM Discover 2.0), equipped with digital temperature control and pressure sensors. PLLA flakes (0.116 g, 1.6 mmol, 160 equiv) were placed in a Pyrex glass tube, followed by the addition of 3.1 mL of nondistilled solvent ([lactyl unit] = 0.5 M). The mixture was stirred for a few minutes. Subsequently, the zinc catalyst (5.8 mg, 10 μmol, 1 equiv) and water (0.14 mL, 5 equiv relative to the ester linkages) were added to the reactor. The reaction tube was sealed with a silicon cap using a pressure monitor unit and stirred by a magnetic stirrer in the system. The temperature was monitored from the bottom of the glass tube using an internal infrared temperature monitor and maintained at desired temperature by adjusting the irradiation power. At this point, the reaction time was initiated, and the decomposition of PLLA was monitored until complete disappearance of the solid phase. After the reaction, the vessel was cooled by blowing compressed air through the slot provided to avoid overheating of the samples during microwave irradiation. The conversion of internal methine units (X_int_), lactic acid selectivity (S_LA_), and lactic acid yield (Y_LA_) were determined by ^1^H NMR spectroscopy.

### 4.2 Hydrolysis of PLLA under Solvent‐Free Conditions

The hydrolysis of PLLA was carried out in a microwave reactor (CEM Discover 2.0), equipped with digital temperature control and pressure sensors. PLLA flakes (0.116 g, 1.6 mmol, 160 equiv), zinc catalyst (5.8 mg, 10 μmol, 1 equiv), and water (0.14 mL, 5 equiv relative to the ester linkages) were placed in a Pyrex glass tube. The reaction tube was sealed with a silicon cap using a pressure monitor unit and stirred by a magnetic stirrer in the system. The temperature was monitored from the bottom of the glass tube using an internal infrared temperature monitor and maintained at desired temperature by adjusting the irradiation power. At this point, the reaction time was initiated, and the decomposition of PLLA was monitored until complete disappearance of the solid phase. After the reaction, the vessel was cooled by blowing compressed air through the slot provided to avoid overheating of the samples during microwave irradiation, and DMSO was added to the mixture to solubilize all residual species and obtain a homogeneous solution. The conversion of internal methine units (X_int_), lactic acid selectivity (S_LA_), and lactic acid yield (Y_LA_) were determined by ^1^H NMR spectroscopy. For comparison, some reactions were carried out with conventional heating using an oil bath.

## Supporting Information

Additional supporting information can be found online in the supporting information section. **Supporting Fig. S1**: ^1^H NMR spectrum (600 MHz, C6D6, 298 K) of ligand LH. **Supporting Fig. S2**: ^1^H NMR spectrum (600 MHz, C_6_D_6_, 298 K) of complex **1**. **Supporting Fig. S3**: ^1^H NMR spectrum (600 MHz, DMSO‐d6, 298 K) of complex **1**. **Supporting Fig. S4**: *COSY* NMR spectrum (600 MHz, DMSO‐d6, 298 K) of complex **1**. **Supporting Fig. S5**: *HSQC* NMR spectrum (600 MHz, DMSO‐d6, 298 K) of complex **1**. **Supporting Fig. S6**: ^13^C NMR spectrum (150 MHz, DMSO‐d6, 298 K) of complex **1**. **Supporting Fig. S7**: ^1^H NMR spectra (600 MHz, DMSO‐d6, 298 K) of complex **1** (a) and complex **1** in the presence of 30 eq of water (b). **Supporting Fig. S8**: ^1^H NMR spectrum (400 MHz, CDCl_3_, 298 K) of commercial PLLA. **Supporting Fig. S9**: GPC trace of commercial PLLA in THF solution. **Supporting Fig. S10**: TGA of commercial PLLA. **Supporting Fig. S11**: DSC thermogram of commercial PLLA. **Supporting Fig. S12**: ^1^H NMR spectrum (400 MHz, DMSO‐d_6_, 298K) showing assignment of degradation products in terms of methyl groups.[3] **Supporting Fig. S13**: ^1^H NMR spectrum (600 MHz, DMSO‐d_6_, 298 K) of PLLA hydrolysis after 1h (entry 4, Table S2) with TMSS standard. **Supporting Fig. S14**: Overlaid chromatographic peaks of lactic acid calibration standards. **Supporting Fig. S15**: Linear regression obtained through HPLC analysis of lactic acid calibration standards. **Supporting Fig. S16**: ^1^H NMR spectrum (400 MHz, CDCl_3_, 298K) of benzyl 2‐hydroxypropanoate. **Supporting Fig. S17**: HPLC Chromatogram of racemic benzyl 2‐hydroxypropanoate obtained from the benzylation of *rac*‐lactic acid. **Supporting Fig. S18**: HPLC Chromatogram of benzyl 2‐hydroxypropanoate obtained from the benzylation of lactic acid formed after hydrolysis of PLLA with **1**, at 130 °C under solvent‐free, microwave‐assisted conditions (entry 1, Table 3). **Supporting Fig. S19**: ^1^H NMR spectrum (400 MHz, DMSO‐d_6_, 298K) of lactic acid. **Supporting Fig. S20**: ^1^H NMR spectrum (600 MHz, DMSO‐d_6_, 298K) of the lactic acid (85%) and dilactic acid (15%) mixture from entry 5, Table 3. **Supporting Fig. S21**: ^1^H NMR spectra (600 MHz, DMSO‐d6, 298K) of: (a) the lactic acid (85%) and dilactic acid (15%) mixture obtained from entry 5, Table 3 (black);  (b) an *ad hoc‐prepared* mixture of lactic acid (43%) and dilactic acid (57%)*;* and (c) commercial lactic acid (red). **Supporting Fig. S22**: Images of the hydrolysis reaction mixture before and after distillation. **Supporting Fig. S23**: ^1^H NMR spectra (600 MHz, DMSO‐d6, 298K) of reaction mixture obtained from entry 8, Table 3; (a) before vacuum distillation; (b) residue after vacuum distillation; and (c) distillate obtained by vacuum distillation. **Supporting Fig. S24**: GPC curves of the starting PLLA (black), PLLA irradiated microwave for 15 minutes (X_int_ = 23%, red), PLLA heated conventionally for 30 minutes (X_int_ = 20 %, blue). **Supporting** **Table** **S1**: Peak areas for lactic acid calibration standards and the analyzed sample. **Supporting** **Table** **S2**: GPC analysis of residual polymer after hydrolysis in solution. **Supporting** **Table** **S3**: GPC analysis of residual polymer after hydrolysis under solvent‐free conditions.

## Author Contributions

**Federica Santulli**: data curation (equal), investigation (equal), writing – original draft (equal), writing – review & editing (equal). **Maëlie Chauvin**: data curation (equal), investigation (equal), writing – review & editing (equal). **Rosaria Schettini**: data curation (equal), investigation (equal), writing – review & editing (equal). **Marina Lamberti**: data curation (equal), formal analysis (equal), writing – review & editing (equal). **Frédéric de Montigny**: data curation (equal), formal analysis (equal), visualization (equal), writing – review & editing (equal). **Christophe M. Thomas**: writing – original draft (equal), writing – review & editing (equal). **Mina Mazzeo**: conceptualization (equal), data curation (equal), formal analysis (equal), writing – original draft (equal), writing – review & editing (equal).

## Funding

MICS (Made in Italy – Circular and Sustainable‐VESTITO project) Extended Partnership and received funding from the European Union Next‐ GeneraFonEU (PIANO NAZIONALE DI RIPRESA E RESILIENZA (PNRR) – MISSIONE 4 COMPONENTE 2, INVESTIMENTO 1.3 – D.D. 1551.11‐10‐2022, PE00000004) (CUP B43C22000740006).

## Conflicts of Interest

The authors declare no conflicts of interest.

## Supporting information

Supplementary Material

## Data Availability

The data that support the findings of this study are available in the supplementary material of this article.
