# Supplementary material for: Efficient Microwave‐Assisted Hydrolytic Recycling of Poly(L‐Lactic Acid)
Source: ChemSusChem. 2026 Feb 26;19(5):e202502366. doi: 10.1002/cssc.202502366 (PMC12936649; doi:10.1002/cssc.202502366)
Supplement: Supplementary file 1 — Supplementary Material [file CSSC-19-e202502366-s001.pdf]

Supporting Information

Efficient Microwave-Assisted Hydrolytic Recycling of Poly(L-lactic acid)

Federica Santulli,*[a] Maëlie Chauvin,[b] Rosaria Schettini,[a] Marina Lamberti,[a] Frédéric de Montigny,[b] Christophe Thomas,*[b] Mina Mazzeo[a]

[a]Department of Chemistry and Biology "Adolfo Zambelli" University of Salerno, via Giovanni Paolo II, 132. 84084 Fisciano (SA) Italy.

[b]Chimie ParisTech, PSL University, CNRS, Institut de Recherche de Chimie Paris, 75005 Paris, France.

Email: fsantulli@unisa.it, christophe.thomas@chimie-paristech.fr

Table of Contents

Experimental section 4

Characterization of the ligand 6

Figure S1. ^{1}H NMR spectrum (600 MHz, C_6D_6, 298 K) of ligand LH 6

Characterization of the complex 6

Figure S2. ^{1}H NMR spectrum (600 MHz, C_6D_6, 298 K) of complex **1**. 6

Figure S3. ^{1}H NMR spectrum (600 MHz, DMSO-d6, 298 K) of complex **1**. 7

Figure S4. *COSY* NMR spectrum (600 MHz, DMSO-d6, 298 K) of complex **1**. 7

Figure S5. *HSQC* NMR spectrum (600 MHz, DMSO-d6, 298 K) of complex **1**. 8

Figure S6. ^{13}C NMR spectrum (150 MHz, DMSO-d6, 298 K) of complex **1**. 8

Figure S7. ^{1}H NMR spectra (600 MHz, DMSO-d6, 298 K) of complex **1** (a) and complex **1** in the presence of 30 eq of water (b). 9

Characterization of starting PLLA 9

Figure S8. ^{1}H NMR spectrum (400 MHz, $CDCl_3$, 298 K) of commercial PLLA. The methine region of the homonuclear decoupled ^{1}H NMR spectrum is shown at the top. Statistical analysis based on the Bernoullian distribution gives a probability of meso dyads (P_m) of 0.98.[1] 9

Figure S9. GPC trace of commercial PLLA in THF solution (1 mg/mL) calibrated against polystyrene standards and corrected using a factor of 0.58, yielding a number-average molecular weight M_n of 51.[2] 10

Figure S10. TGA of commercial PLLA. 10

Figure S11. DSC thermogram of commercial PLLA, recorded during the second heating scan. 11

Quantitative Analysis of Degradation Products 12

NMR analysis 12

Figure S12. ^{1}H NMR spectrum (400 MHz, DMSO-d_6, 298K) showing assignment of degradation products in terms of methyl groups. [3] 12

Figure S13. ^{1}H NMR spectrum (600 MHz, DMSO-d_6, 298 K) of PLLA hydrolysis after 1h (entry 4, Table S2) with TMSS standard. 12

Quantitative reverse HPLC analysis 13

Figure S14. Overlaid chromatographic peaks of lactic acid calibration standards. 13

Figure S15. Linear regression obtained through HPLC analysis of lactic acid calibration standards. 13

Table S1. Peak areas for lactic acid calibration standards and the analyzed sample. 14

Hydrolysis of PLLA in solution 15

Table S2. GPC analysis of residual polymer after hydrolysis in solution. 15

Synthesis and chiral analysis of benzyl 2-hydroxypropanoate (benzyl lactate) 16

Synthesis of benzyl 2-hydroxypropanoate 16

Figure S16. ^{1}H NMR spectrum (400 MHz, $CDCl_3$, 298K) of benzyl 2-hydroxypropanoate. 16

Chiral HPLC analysis 17

Figure S17. HPLC Chromatogram of racemic benzyl 2-hydroxypropanoate obtained from the benzylation of *rac*-lactic acid. 17

Figure S18. HPLC Chromatogram of benzyl 2-hydroxypropanoate obtained from the benzylation of lactic acid formed after hydrolysis of PLLA with **1**, at 130 °C under solvent-free, microwave-assisted conditions (entry 1, Table 3). 17

Hydrolysis of PLLA under solvent-free conditions 18

Table S3. GPC analysis of residual polymer after hydrolysis under solvent-free conditions. 18

Figure S19. ^{1}H NMR spectrum (400 MHz, DMSO-d_6, 298K) of lactic acid. 18

Figure S20. ^{1}H NMR spectrum (600 MHz, DMSO-d_6, 298K) of the lactic acid (85%) and dilactic acid (15%) mixture from entry 5, Table 3. 19

Figure S21. ^{1}H NMR spectra (600 MHz, DMSO-d_6, 298K) of: (a) the lactic acid (85%) and dilactic acid (15%) mixture obtained from entry 5, Table 3 (black); (b) an *ad hoc-prepared* mixture of lactic acid (43%) and dilactic acid (57%)*;* and (c) commercial lactic acid (red). 19

Purification of the hydrolysis product by vacuum distillation 20

Figure S22. Images of the hydrolysis reaction mixture before and after distillation. 20

Figure S23. ^{1}H NMR spectra (600 MHz, DMSO-d_6, 298K) of reaction mixture obtained from entry 8, Table 3: (a) before vacuum distillation (lactic acid-to-dilactic acid molar ratio 85:15); (b) residue after vacuum distillation (30% of starting mixture, lactic acid-to-dilactic acid molar ratio 78:22); and (c) distillate obtained by vacuum distillation (70% of starting mixture, lactic acid-to-dilactic acid molar ratio 95:5). 20

Figure S24. GPC curves of the starting PLLA (black), PLLA irradiated microwave for 15 minutes (X_{int} = 23%, red), PLLA heated conventionally for 30 minutes (X_{int} = 20 %, blue). 21

References 21

Experimental section

Reagents and solvents

PLLA waste cups (Natura Bio, Aristea S.p.A. or Selex S.p.A.) were used as received and mechanically cut into small pieces prior to use. N,N-Dimethylformamide (DMF, > 99%), dimethyl sulfoxide (DMSO, > 99.9%), 1,3-dioxolane (> 99%), 2-methyltetrahydrofuran (MeTHF, > 99%), anisole (99.7 %) and acetone (> 99.5%) were obtained from Merck or Carlo Erba and used as received without further purification unless otherwise stated. Deionized water was used as the reagent in the hydrolysis reactions.

NMR analysis

NMR spectra were recorded on Bruker Avance 300, 400 and 600 MHz spectrometers at 25 °C. Chemical shift (δ) are reported as parts per million (ppm) and coupling constants (J) in hertz. ^{1}H NMR spectra are referenced using the residual solvent peak at δ = 7.16 for C_6D_6, δ = 7.26 for $CDCl_3$, and δ = 2.50 for DMSO-d_6. ^{13}C NMR spectra are referenced using the residual solvent peak at δ = 39.52 for DMSO-d_6.

Microwave reactor

All the microwave assisted reactions have been performed using the CEM reactor model Discover 2.0. The reactor can work with 10 and 35 mL glass pressurized vessels and is equipped with an IR temperature sensor and a touchscreen interface from which is possible to program the parameters of the reaction such as temperature, time, stirring power, power limit that the instrument can reach and max pressure that the reactor can handle before activating the safety control of pressure and release all the excess gasses.

GPC analysis

Molecular masses (M_n) and their dispersities (M_w/M_n) were measured by gel permeation chromatography (GPC). The measurements were performed by an Infinity II-Agilent (Santa Clara, CA, USA) system equipped with a refractive index detector. Tetrahydrofuran (THF) was used as eluent at 35 °C at a flow rate of 1.0 mL min^{-1}. The calibration curve was obtained using 26 polystyrene standards covering a molecular weight range from 370 to 1,210,000 Da. The analyzed samples were dissolved in THF with a concentration of 1 mg mL^{-1}. The solutions obtained were then filtered using a Chromafil PTFE 0.45 μm filter.

DSC analysis

Glass transition temperatures (T_g), melting points (T_m) and enthalpy of fusion (ΔH_m) of the polymer samples were measured by differential scanning calorimetry (DSC) using aluminum pans and a DSC 2920 TA Instruments apparatus, calibrated with indium. Measurements were performed under nitrogen flow with a heating rate of 10°C min^{-1} in the range opportune range of temperature. DSC data were processed with TA Universal Analysis v2.3.

HPLC for quantitative analysis

Analytical HPLC was done on JASCO LC-NET II/ADC equipped with JASCO PU-4180 RHPLC Pump and a diode-array detector JASCO MD-4010 and extracted wavelengths of 220 nm. LC systems used a linear gradient of solvent B (acetonitrile with 0.1% TFA) in solvent A (solvent water with 0.1% TFA) run over 30 min, flow rate 1.0 mL/min, and UV absorption at 220 nm. Reversed-phase analysis was done on a C18 column, Waters, Bondapak, 10 μm, 125 Å, 3.9 mm × 300 mm.

HPLC for chiral analysis

HPLC analyses were performed on a Jasco LC-NET II/ADC equipped with a Jasco Model PU-2089 Plus Pump and a Jasco MD-2010 Plus UV-vis multiple wavelength detector. Enantiomeric excesses of products were determined by chiral HPLC using Chiralcel OD-H columns with an UV detector set at 220 nm.

Characterization of the ligand

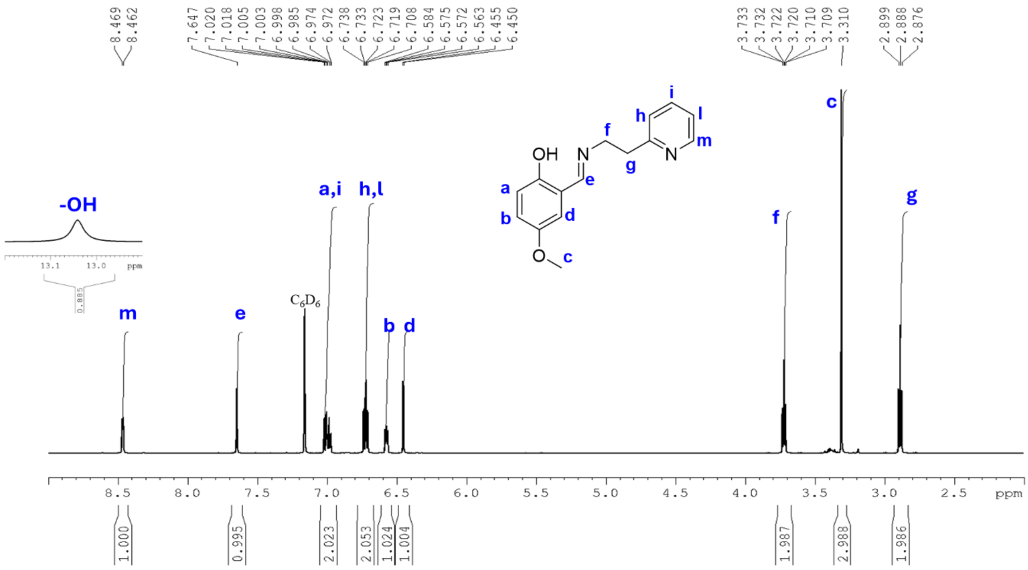

Figure S1. ^1H NMR spectrum (600 MHz, C_6D_6, 298 K) of ligand LH.

^1H NMR (600 MHz, C_6D_6, 298 K): δ 13.04 (br, 1H, -OH), 8.46 (d, J = 4.2 Hz, 1H, Hm), 7.65 (s, 1H, He), 7.00 (m, 2H, Ha+Hi), 6.73 (m, 2H, Hh+Hl), 6.57 (t, J = 5.4 Hz, 1H, Hb), 6.45 (d, J = 3.0 Hz, 1H, Hd), 3.72 (t, J = 7.2 Hz, 2H, Hf), 3.10 (s, 3H, Hc), 2.89 (t, J = 7.2 Hz, 2H, Hg).

Characterization of the complex

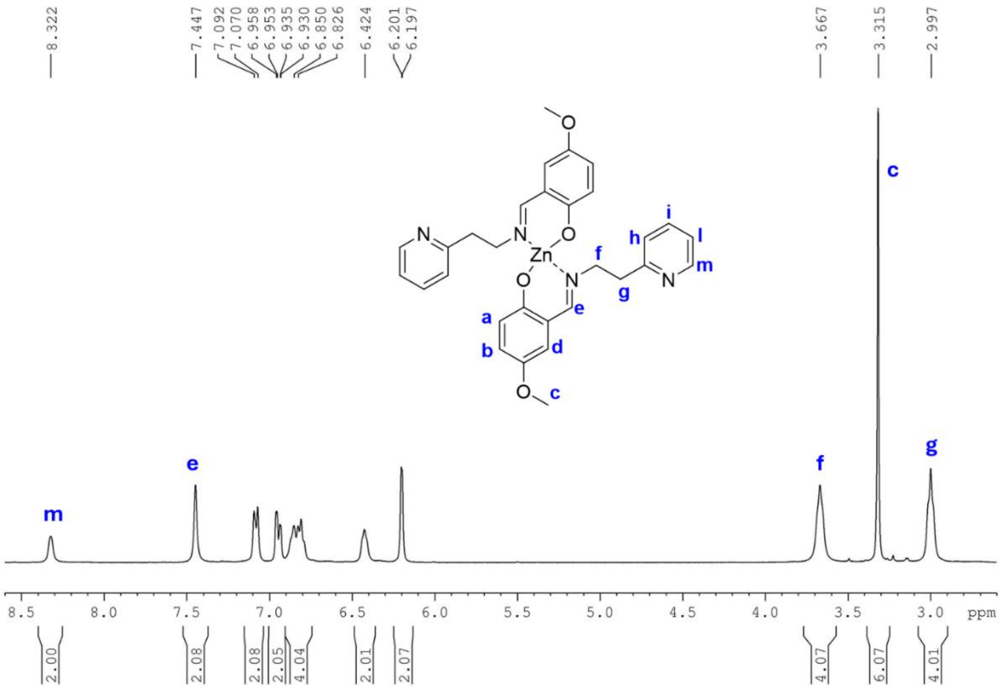

Figure S2. ^1H NMR spectrum (600 MHz, C_6D_6, 298 K) of complex **1**.

^1H NMR (600 MHz, C_6D_6, 298 K): δ 8.46 (br, 2H, Hm), 7.45 (s, 2H, He), 7.08 (d, J = 8.8 Hz, 2H, Ar), 6.95 (d, J = 7.2, 2H, Ar), 6.83 (m, 4H, Ar), 6.42 (br, 2H, Ar), 6.20 (s, 2H, Ar), 3.67 (br, 4H, Hf), 3.31 (s, 6H, Hc), 3.00 (br, 4H, Hg).

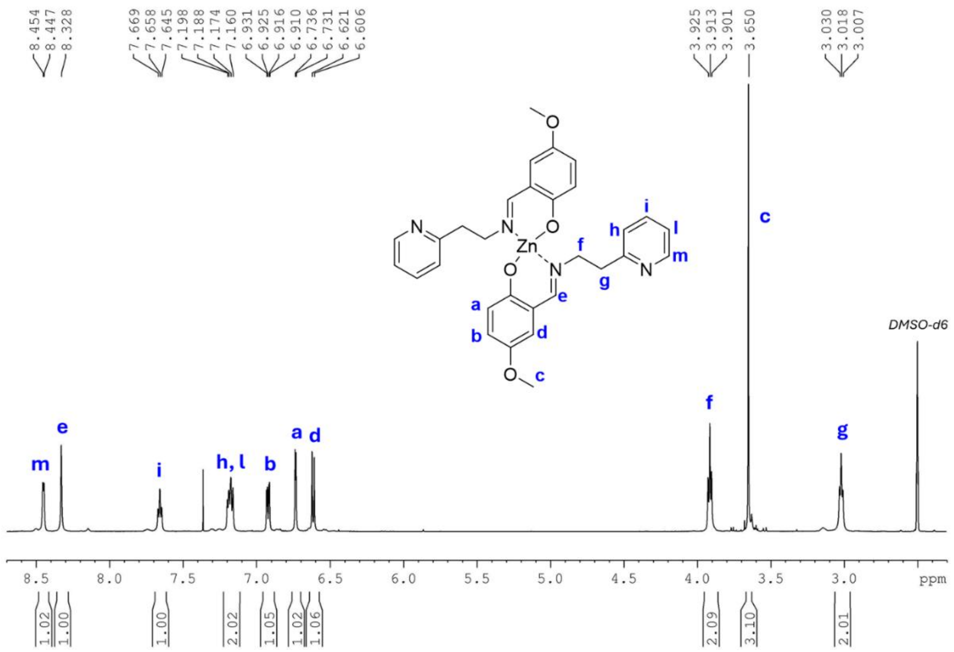

Figure S3. ^{1}H NMR spectrum (600 MHz, DMSO-d6, 298 K) of complex **1**.

^{1}H NMR (600 MHz, DMSO-d6, 298 K): δ 8.45 (d, J = 4.2 Hz, 2H, Hm), 8.33 (s, 2H, He), 7.65 (t, J = 6.6 Hz, 2H, Hi), 7.18 (m, 4H, Hh+Hl), 6.92 (dd, J_1 = 9.0 Hz, J_2 = 3.6 Hz, 2H, Hb), 6.73 (d, J = 3.6 Hz, 2H, Ha), 6.61 (d, J = 9.0, 2H, Hd), 3.91 (t, J = 7.2 Hz, 4H, Hf), 3.65 (s, 6H, Hc), 3.02 (t, J = 7.2 Hz, 4H, Hg).

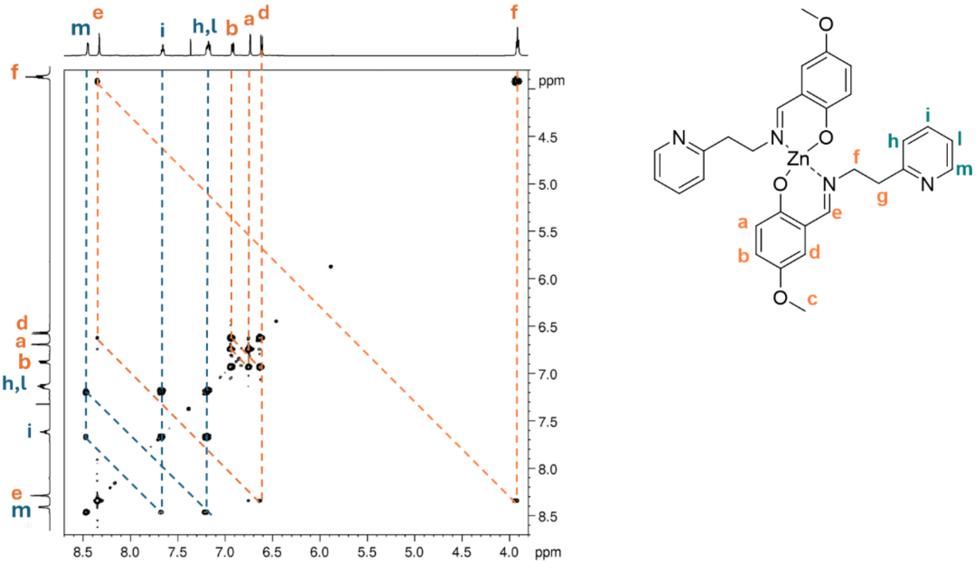

Figure S4. *COSY* NMR spectrum (600 MHz, DMSO-d6, 298 K) of complex **1**.

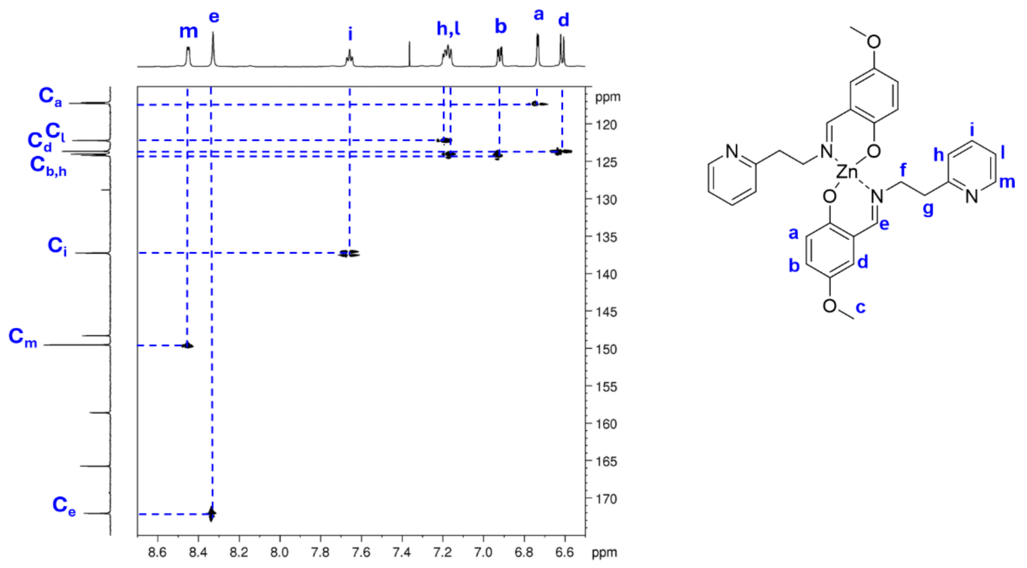

Figure S5. *HSQC* NMR spectrum (600 MHz, DMSO-d6, 298 K) of complex **1**.

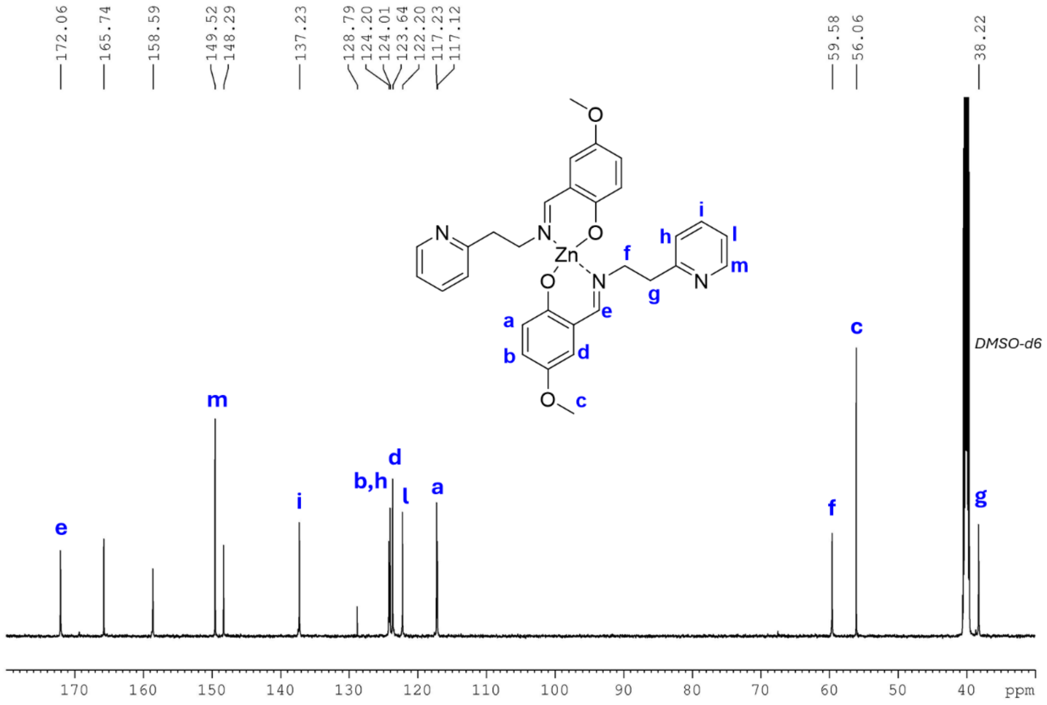

Figure S6. ^{13}C NMR spectrum (150 MHz, DMSO-d6, 298 K) of complex **1**.

^{13}C NMR (150 MHz, DMSO-d6, 298 K): δ 172.1 (Ce), 165.7 (C*q*), 158.6 (C*q*), 149.5 (Cm), 148.3 (C*q*), 137.2 (Ci), 128.8 (Cb), 124.2 (Ch), 123.6 (Cd), 122.2 (Cl), 117.2 (Ca), 59.6 (Cf), 56.1 (Cc), 38.2 (Cg).

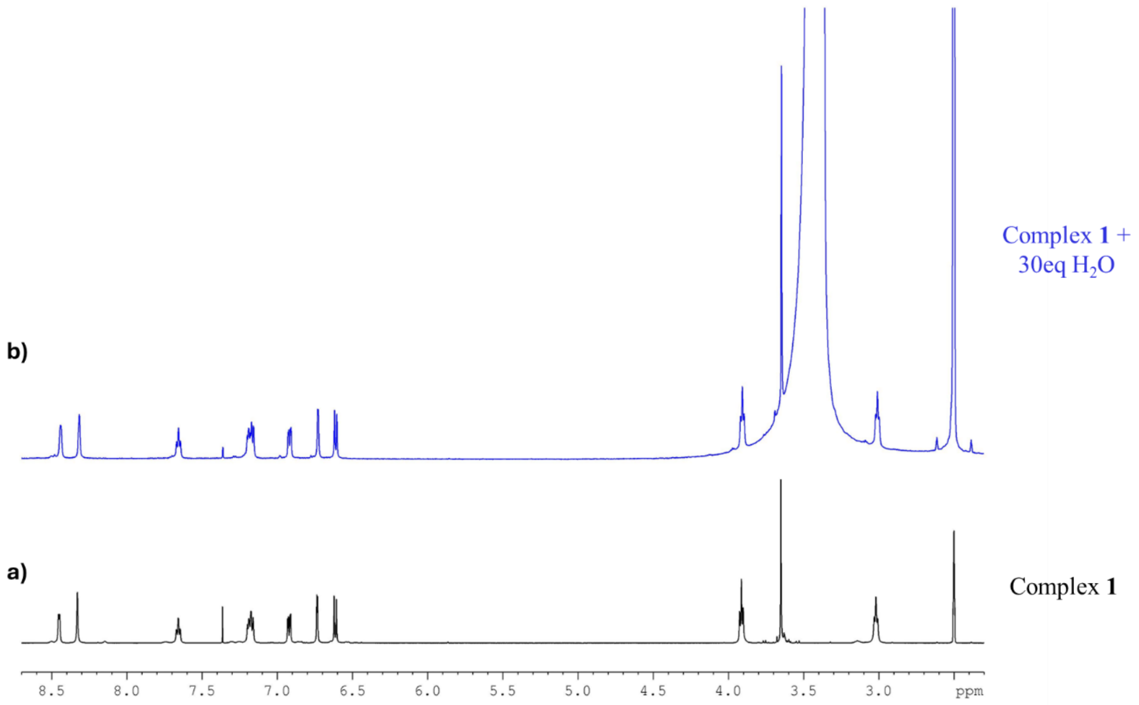

Figure S7. ^{1}H NMR spectra (600 MHz, DMSO-d6, 298 K) of complex **1** (a) and complex **1** in the presence of 30 eq of water (b).

Characterization of starting PLLA

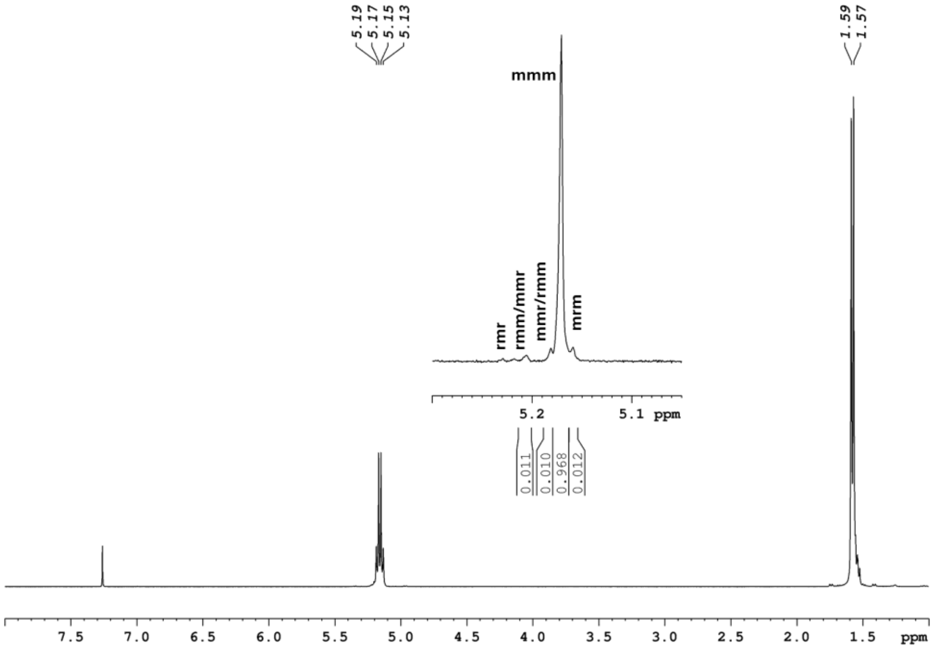

Figure S8. ^{1}H NMR spectrum (400 MHz, $CDCl_3$, 298 K) of commercial PLLA. The methine region of the homonuclear decoupled ^{1}H NMR spectrum is shown at the top. Statistical analysis based on the Bernoullian distribution gives a probability of meso dyads (P_m) of 0.98.[1]

Molecular Weight Averages

Peak	Mp (g/mol)	Mn (g/mol)	Mw (g/mol)	Mz (g/mol)	Mz+1 (g/mol)	Mv (g/mol)
Peak 1	131791	87832	165837	259445	355126	153406

PD
1.888

Distribution Plot

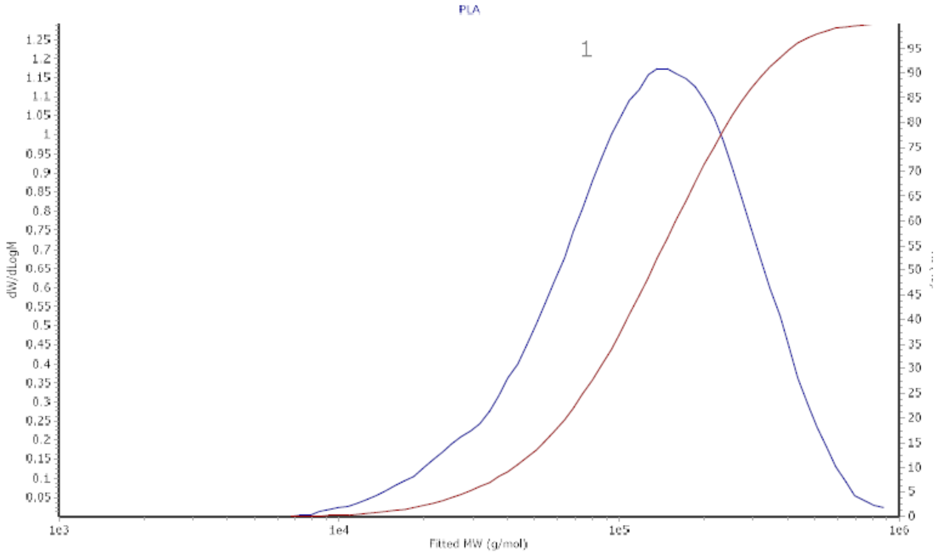

Figure S9. GPC trace of commercial PLLA in THF solution (1 mg/mL) calibrated against polystyrene standards and corrected using a factor of 0.58, yielding a number-average molecular weight M_n of 51.[2]

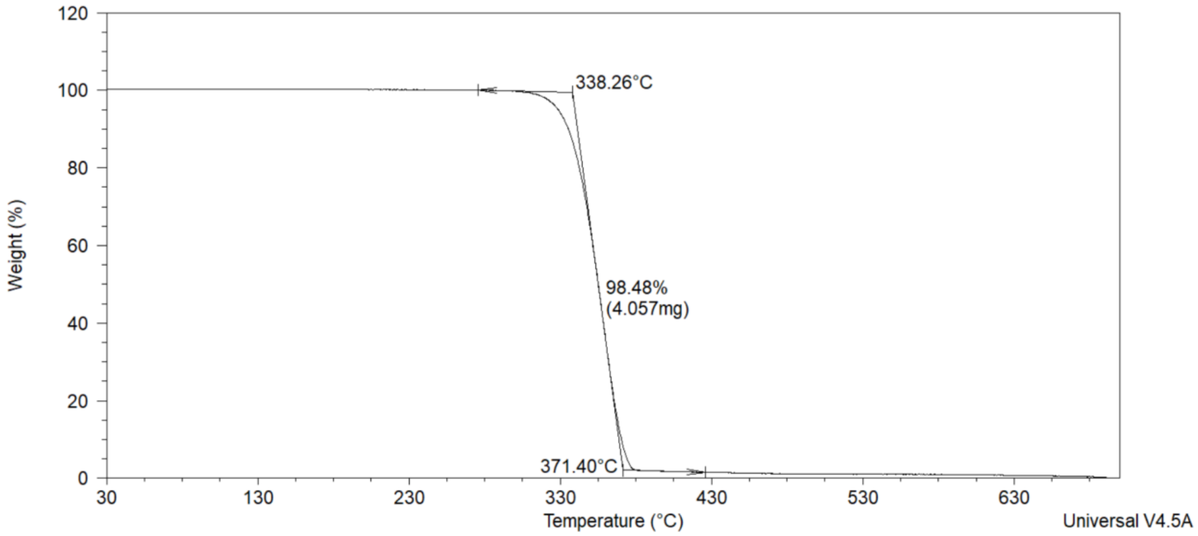

Figure S10. TGA of commercial PLLA.

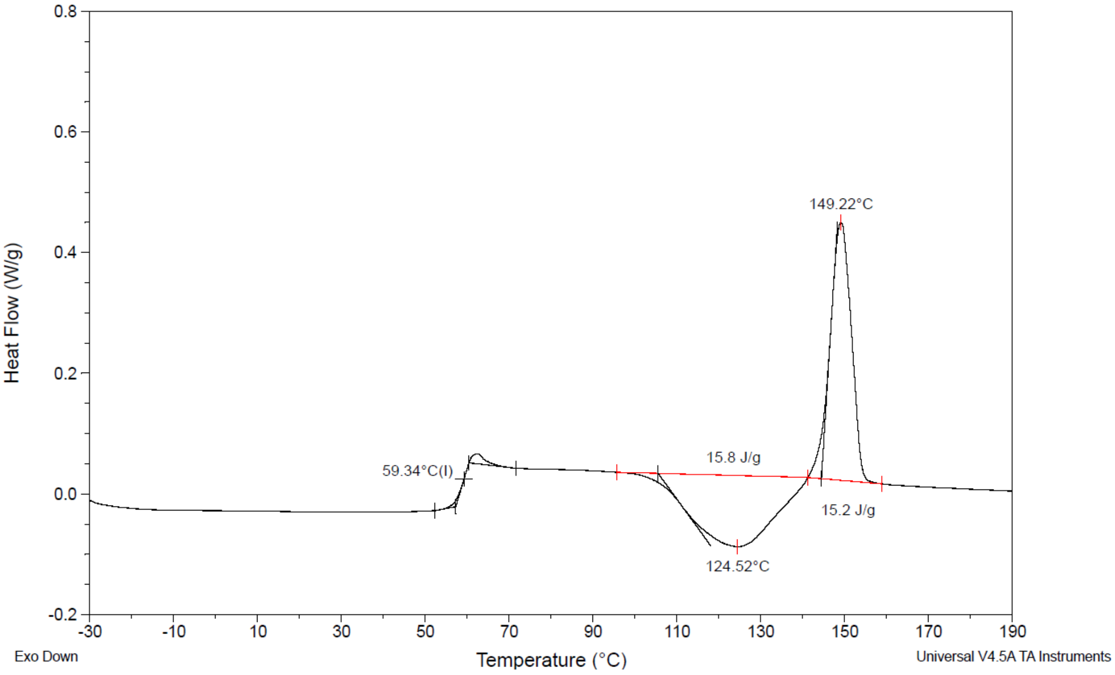

Figure S11. DSC thermogram of commercial PLLA, recorded during the second heating scan.

Quantitative Analysis of Degradation Products

NMR analysis

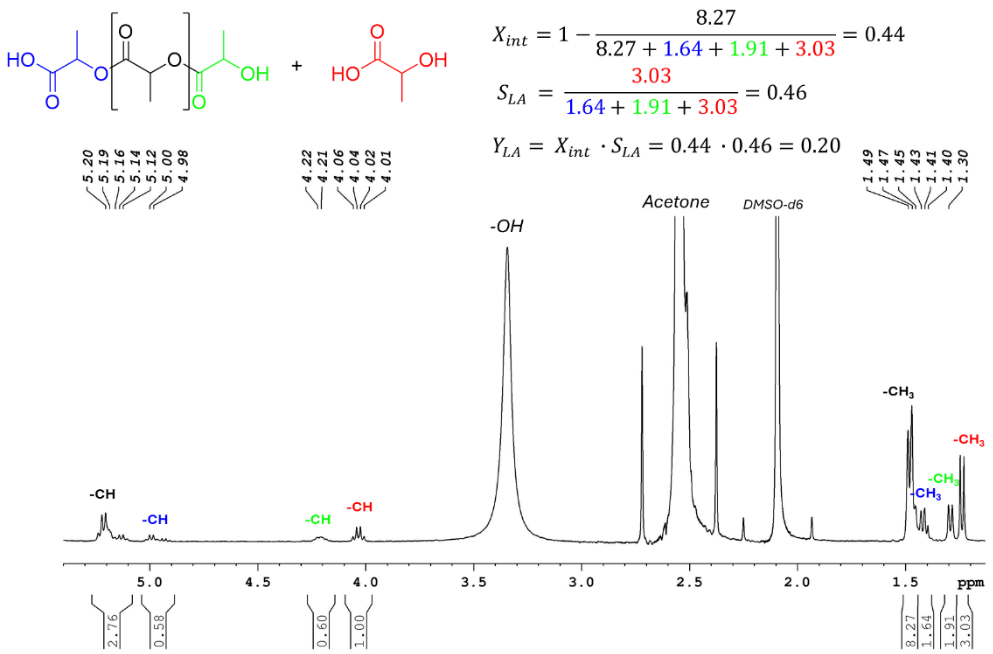

Figure S12. ^{1}H NMR spectrum (400 MHz, DMSO-d$_6$, 298K) showing assignment of degradation products in terms of methyl groups. [3]

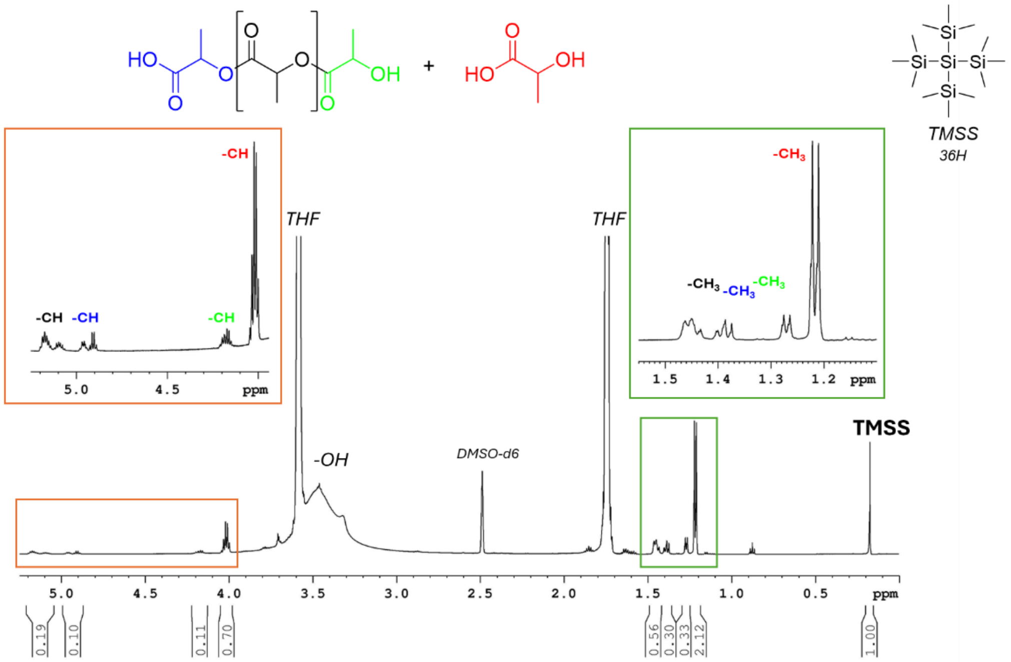

Figure S13. ^{1}H NMR spectrum (600 MHz, DMSO-d$_6$, 298 K) of PLLA hydrolysis after 1h (entry 4, Table S2) with TMSS standard.

Reaction condition: 10 µmol of 1 (0.6 mol % relative to ester linkages) 0.116 g of PLLA from transparent cup, 0.42 mL of H_2O (15 eq. with respect to the lactyl units), TMSS (0.013g, 0.042 mmol) under solvent-free condition using microwave reactor. After the reaction, THF was added to solubilize all the product.

Ester linkages: TMSS [36:1],

$$Y_{La} = \frac{[La_{CH_3}]}{3} \cdot \frac{1}{[TMSS]} \cdot 100 = \frac{2.12}{3} \cdot \frac{1}{1} \cdot 100 = 70\ \%$$

$$X_{int} = 1 - \frac{[PLA_{CH_3}]}{3} \cdot \frac{1}{[TMSS]} \cdot 100 = 1 - \frac{0.56}{3} \cdot \frac{1}{1} \cdot 100 = 81\ \%$$

Quantitative reverse HPLC analysis

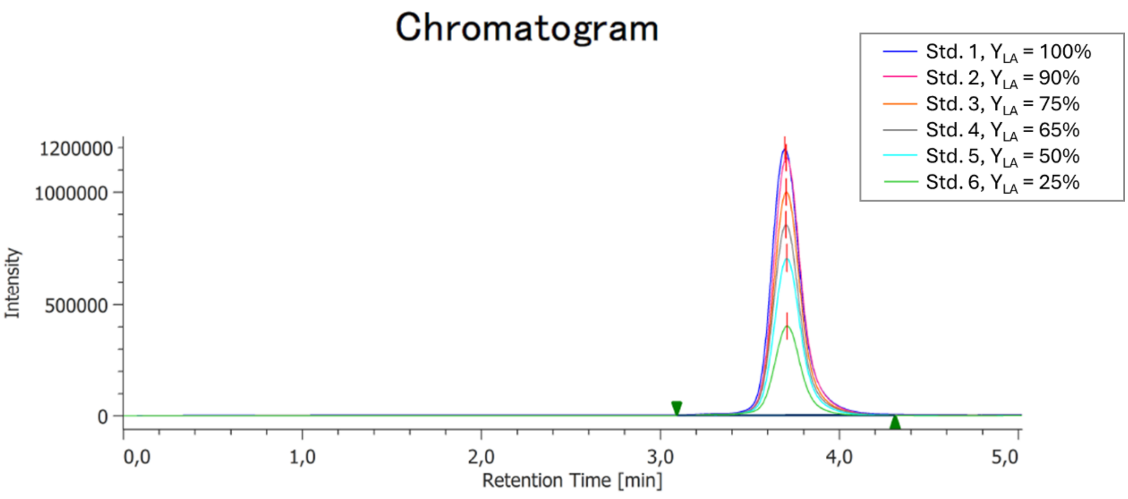

Figure S14. Overlaid chromatographic peaks of lactic acid calibration standards.

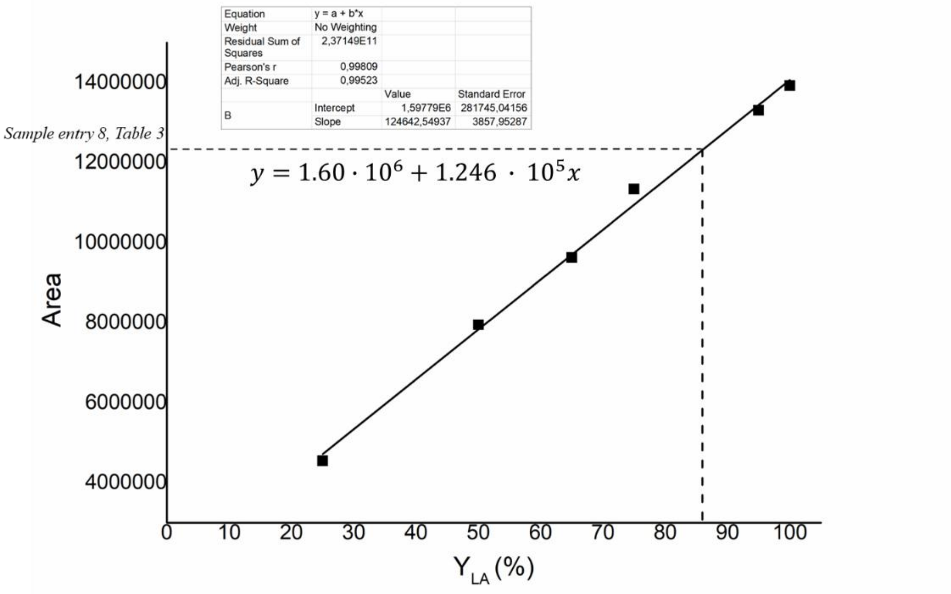

Figure S15. Linear regression obtained through HPLC analysis of lactic acid calibration standards.

Table S1. Peak areas for lactic acid calibration standards and the analyzed sample.

Sample type	Concentration (M)	Y_{LA} (%)	Area
Std. 1	4.2	100	13926366
Std. 2	3.8	90	13301644
Std. 3	3.2	75	11338323
Std. 4	2.7	65	9631172
Std. 5	2.1	50	7945799
Std. 6	1.1	25	4546874
*Sample entry 8, Table 3	3.6	86	12288720

*The sample was prepared by dilution of the crude reaction mixture. Given an initial lactic acid concentration of 6.0 M (Y_{LA} = 100 %), 0.7 mL of crude was diluted to 1.0 mL total volume, resulting in a final lactic acid concentration of 4.2 M.

Channel & Peak Information Table

Chromatogram Name Slz Standard 100
Sample Name
Channel Name 220,0nm

#	Peak Name	CH	tR [min]	Area [µV·sec]	Height [µV]	Area%	Height%	Quantity	NTP	Resolution	Symmetry Factor	Warning
1	Unknown	5	3,69	13926366	1188530	100	100,000	N/A	2612	N/A	1,28	

Chromatogram Name Slz Standard 90
Sample Name
Channel Name 220,0nm

#	Peak Name	CH	tR [min]	Area [µV·sec]	Height [µV]	Area%	Height%	Quantity	NTP	Resolution	Symmetry Factor	Warning
1	Unknown	5	3,70	13301644	1152442	100	100,000	N/A	2708	N/A	1,27	

Chromatogram Name Slz Standard 75
Sample Name
Channel Name 220,0nm

#	Peak Name	CH	tR [min]	Area [µV·sec]	Height [µV]	Area%	Height%	Quantity	NTP	Resolution	Symmetry Factor	Warning
1	Unknown	5	3,70	11338323	999023	100	100,000	N/A	2818	N/A	1,24	

Chromatogram Name Slz Standard 65
Sample Name
Channel Name 220,0nm

#	Peak Name	CH	tR [min]	Area [µV·sec]	Height [µV]	Area%	Height%	Quantity	NTP	Resolution	Symmetry Factor	Warning
1	Unknown	5	3,70	9631172	849317	100	100,000	N/A	2820	N/A	1,23	

Chromatogram Name Slz Standard 50
Sample Name
Channel Name 220,0nm

#	Peak Name	CH	tR [min]	Area [µV·sec]	Height [µV]	Area%	Height%	Quantity	NTP	Resolution	Symmetry Factor	Warning
1	Unknown	5	3,71	7945799	706017	100	100,000	N/A	2907	N/A	1,20	

Chromatogram Name Slz Standard 25
Sample Name
Channel Name 220,0nm

#	Peak Name	CH	tR [min]	Area [µV·sec]	Height [µV]	Area%	Height%	Quantity	NTP	Resolution	Symmetry Factor	Warning
1	Unknown	5	3,71	4546874	403096	100	100,000	N/A	2939	N/A	1,12	

Hydrolysis of PLLA in solution

Table S2. GPC analysis of residual polymer after hydrolysis in solution.

[a]Entry	Solvent	[b]X_{int} (%)	[b]S_{LA} (%)	[b]Y_{LA} (%)	[c]$M_{n,GPC}$ (kDa)	[c]Đ	[d]$M_{n,NMR}$ (kDa)
4, Table 1	1,3-dioxolane	40	40	16	0.75	1.53	0.43
5, Table 1	MeTHF	55	46	34	0.36	1.58	0.32
1, Table 2	Acetone	21	25	5	0.95	1.70	0.76
2, Table 2	Acetone	41	49	20	0.30	2.11	0.57
3, Table 2	Acetone	49	60	29	0.32	1.92	0.48
4, Table 2	Acetone	86	74	64	0.28	1.24	0.30

[a]All reactions were carried out in air by using 10 µmol of **1** (0.6x mol % relative to ester linkages) 0.116 g of PLLA from transparent cup, 0.14 mL of H_2O (5 eq. with respect to the lactyl units) in 3.1 mL of solvent using microwave reactor. [b]Determined by ^{1}H NMR spectroscopy using the equations in Figure 1. [c]Determined by GPC in THF against polystyrene standards and corrected using the factor 0.58. [d]Determined by ^{1}H NMR through integration of the signal at 5.16 ppm (internal methine 1H, q, J = 6.8 Hz) relative to the signal at 4.22 ppm (external methine 1H, q, J = 6.8 Hz) (400 MHz, 294 K, DMSO) as reported in the literature.[4]

Synthesis and chiral analysis of benzyl 2-hydroxypropanoate (benzyl lactate)

Synthesis of benzyl 2-hydroxypropanoate

The procedure was adapted from literature.[5] In a 10 mL vial, lactic acid (0.595 mmol) was added to a solution of triethylamine (60.2 mg, 0.595 mmol) in acetonitrile (2 mL). The mixture was stirred at 60 °C for 30 min. Benzyl bromide (71 µL, 0.595 mmol) was then added, and the reaction was allowed to proceed at 60 °C for 3.5 h. After this time, the solvent was evaporated under reduced pressure. The crude residue was extracted three times with ethyl acetate and purified by silica gel column chromatography (ethyl acetate/hexane 4:6 v/v). The product was obtained as a colorless oil (~ 60 % yield).

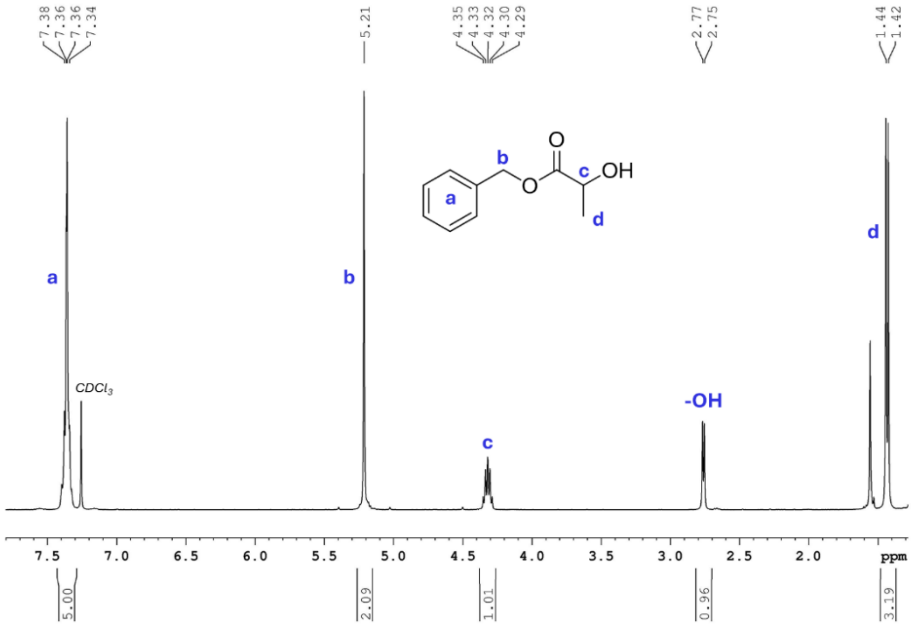

Figure S16. ^{1}H NMR spectrum (400 MHz, $CDCl_3$, 298K) of benzyl 2-hydroxypropanoate.
^{1}H NMR (400 MHz, $CDCl_3$, 298 K): δ 7.36 (m, 5H, Ha), 5.21 (s, 2H, Hb), 4.32 (q, J = 6.2 Hz, 1H, Hc), 2.76 (d, J = 5.3 Hz, -OH), 1.23 (d, J = 6.8 Hz, 3H, Hd).

Chiral HPLC analysis

The product's enantiomeric purity was analyzed by HPLC on a Chiralcel OD-H column, using a mobile phase of hexane/isopropanol (98:2), with a flow rate of 0.5 mL/min, injection volume of 10 µL, and detection at 220 nm (UV).[5]

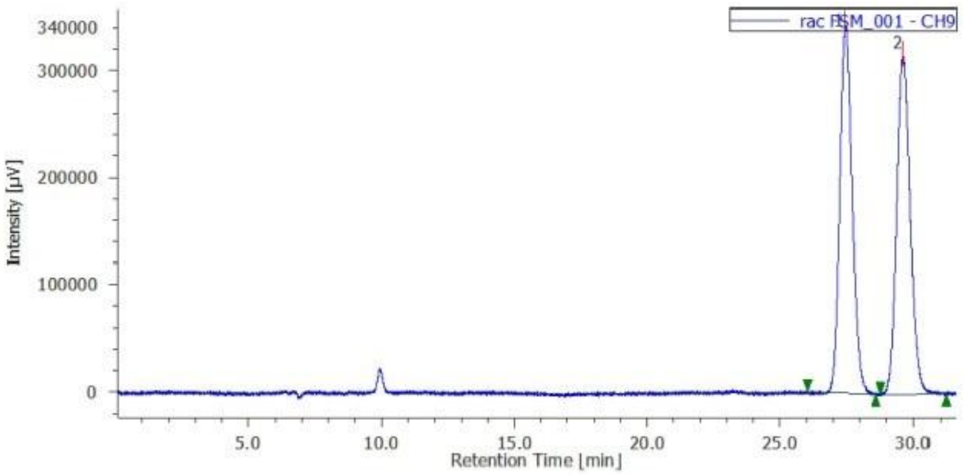

#	Peak Name	CH	tR [min]	Area [µV·sec]	Height [µV]	Area%	Height%	Quantity	NTP	Resolution	Symmetry Factor	Warning
1	Unknown	9	27.438	11114942	344959	49.767	52.022	N/A	16929	2.477	1.202	
2	Unknown	9	29.622	11219008	318142	50.233	47.978	N/A	16436	N/A	1.126	

Figure S17. HPLC Chromatogram of racemic benzyl 2-hydroxypropanoate obtained from the benzylation of *rac*-lactic acid.

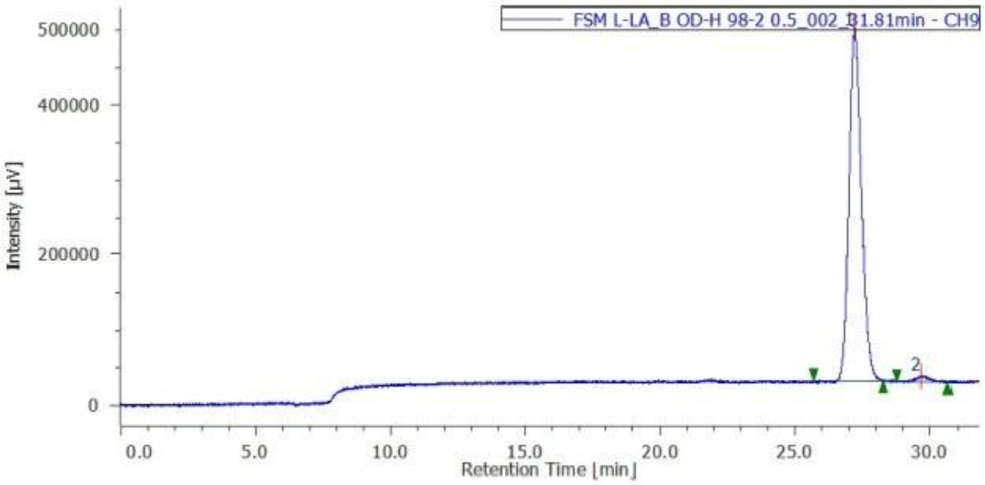

#	Peak Name	CH	tR [min]	Area [µV·sec]	Height [µV]	Area%	Height%	Quantity	NTP	Resolution	Symmetry Factor	Warning
1	Unknown	9	27.222	14874021	471234	97.996	98.170	N/A	17267	2.571	1.181	
2	Unknown	9	29.704	304239	8785	2.004	1.830	N/A	11522	N/A	1.019	

Figure S18. HPLC Chromatogram of benzyl 2-hydroxypropanoate obtained from the benzylation of lactic acid formed after hydrolysis of PLLA with **1**, at 130 °C under solvent-free, microwave-assisted conditions (entry 1, Table 3).

Hydrolysis of PLLA under solvent-free conditions

Table S3. GPC analysis of residual polymer after hydrolysis under solvent-free conditions.

[a]Entry	time (h)	[b]X_{int} (%)	[b]S_{LA} (%)	[b]Y_{LA} (%)	[c]$M_{n,GPC}$ (kDa)	[c]Đ	[d]$M_{n,NMR}$ (kDa)
1	0.25	23	70	16	1.5	1.90	0.88
2	0.50	40	72	29	1.1	1.44	0.75
3	0.75	61	78	47	0.79	1.35	0.50
4	1.0	83	84	70	0.76	1.26	0.34
5	2.0	100	98	98	-	-	-
6	0.50	20	70	14	35	1.45	-

[a]All reactions were carried out in air by using 10 µmol of 1 (0.6 mol % relative to ester linkages) 0.116 g of PLLA from transparent cup, 0.42 mL of H_2O (15 eq. with respect to the lactyl units) under solvent-free condition using microwave reactor. [b]Determined by ^1H NMR spectroscopy using the equations in Figure 1. [c]Determined by GPC in THF against polystyrene standards and corrected using the factor 0.58. [d]Determined by ^1H NMR through integration of the signal at 5.16 ppm (internal methine 1H, q, J = 6.8 Hz) relative to the signal at 4.22 ppm (external methine 1H, q, J = 6.8 Hz) (400 MHz, 294 K, DMSO) as reported in the literature.[4]

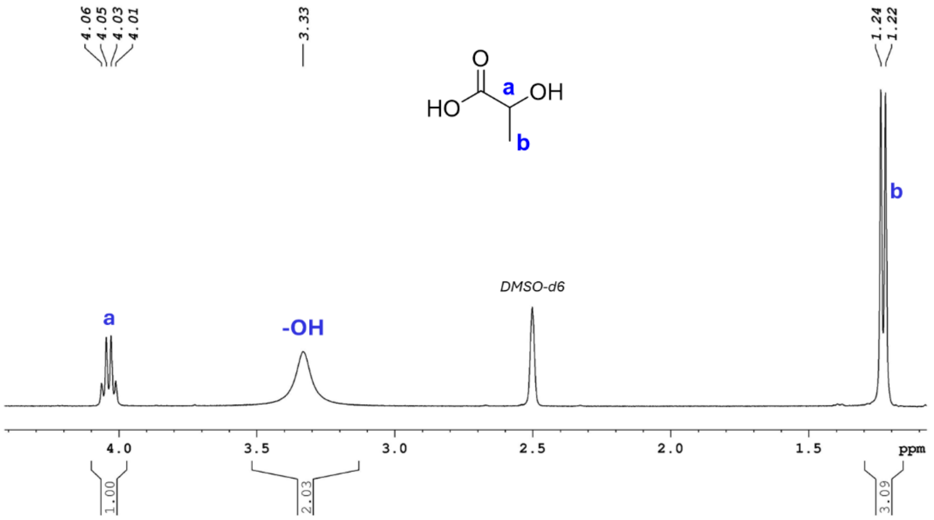

Figure S19. ^1H NMR spectrum (400 MHz, DMSO-d_6, 298K) of lactic acid.
^1H NMR (400 MHz, DMSO-d_6, 298 K): δ 4.04 (q, J = 6.8 Hz, 1H, Ha), 3.33 (br, 2H, -OH), 1.23 (d, J = 6.8 Hz, Hb).

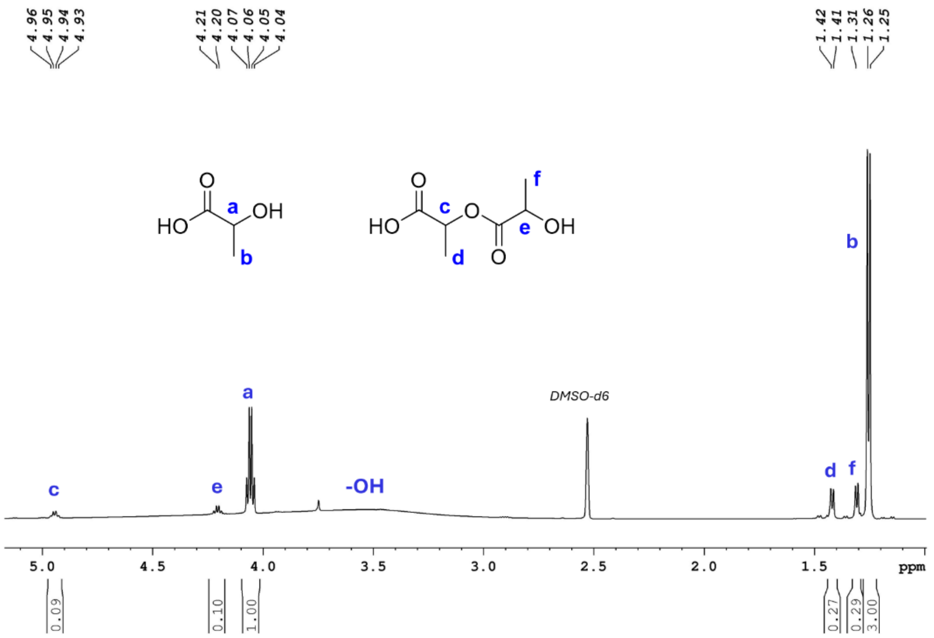

Figure S20. ^{1}H NMR spectrum (600 MHz, DMSO-d_6, 298K) of the lactic acid (85%) and dilactic acid (15%) mixture from entry 5, Table 3.

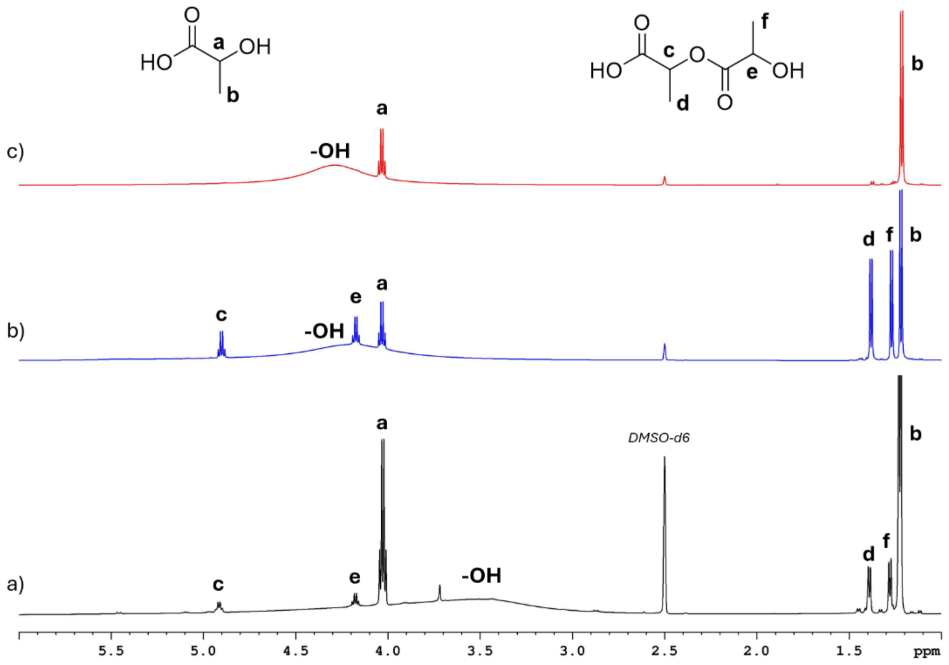

Figure S21. ^{1}H NMR spectra (600 MHz, DMSO-d_6, 298K) of: (a) the lactic acid (85%) and dilactic acid (15%) mixture obtained from entry 5, Table 3 (black); (b) an *ad hoc-prepared* mixture of lactic acid (43%) and dilactic acid (57%)*;* and (c) commercial lactic acid (red).

Purification of the hydrolysis product by vacuum distillation

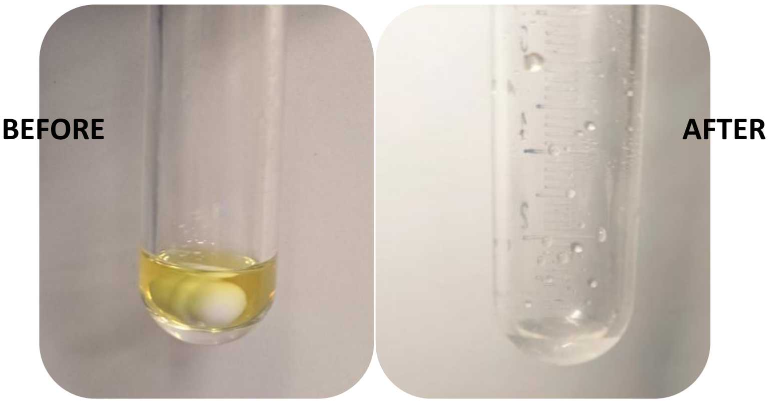

Figure S22. Images of the hydrolysis reaction mixture before and after distillation.

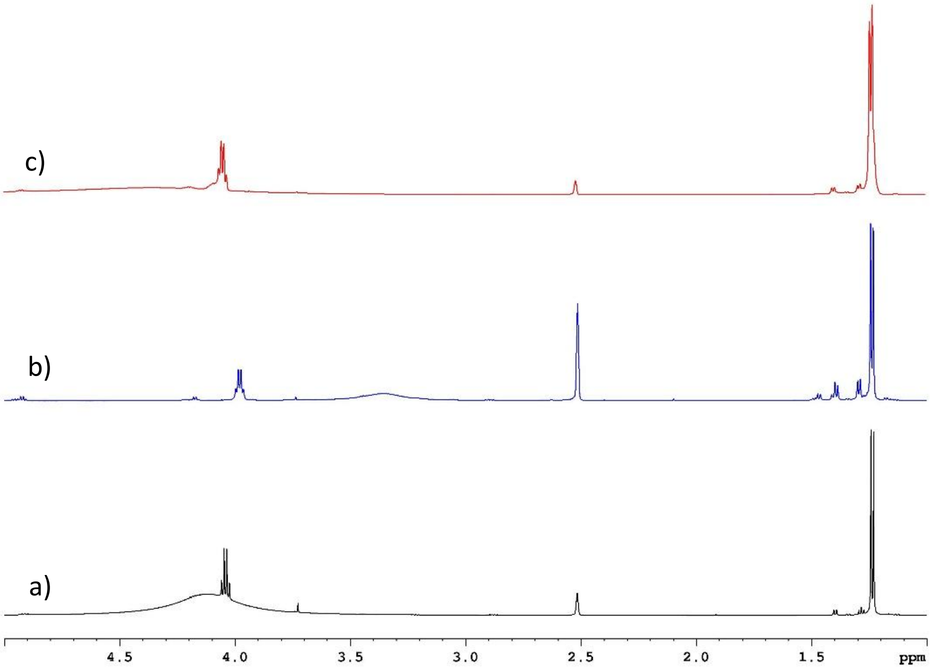

Figure S23. ^{1}H NMR spectra (600 MHz, DMSO-d_6, 298K) of reaction mixture obtained from entry 8, Table 3: (a) before vacuum distillation (lactic acid-to-dilactic acid molar ratio 85:15); (b) residue after vacuum distillation (30% of starting mixture, lactic acid-to-dilactic acid molar ratio 78:22); and (c) distillate obtained by vacuum distillation (70% of starting mixture, lactic acid-to-dilactic acid molar ratio 95:5).

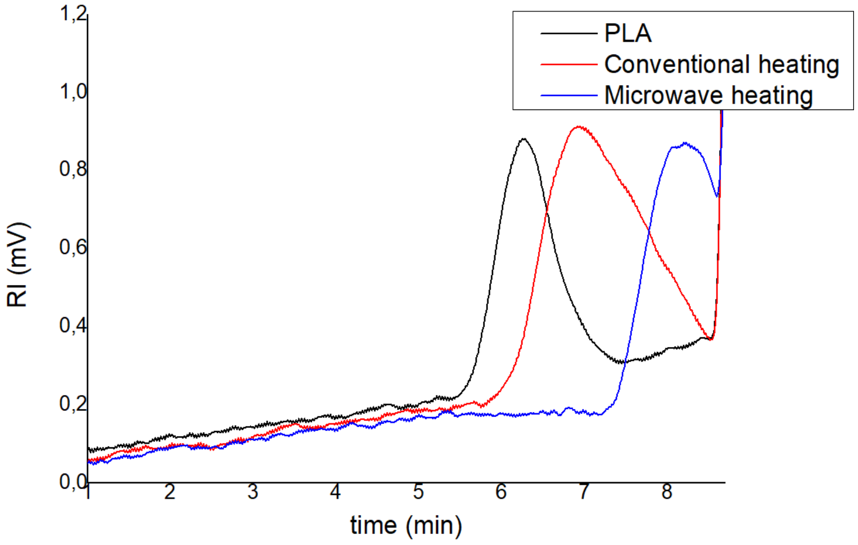

Figure S24. GPC curves of the starting PLLA (black), PLLA irradiated microwave for 15 minutes (X_{int} = 23%, red), PLLA heated conventionally for 30 minutes (X_{int} = 20 %, blue).

References

[1] T. M. Ovitt, G. W. Coates, *J. Am. Chem. Soc.* **2002**, *124*, 1316-1326.

[2] M. Save, M. Schappacher, A. Soum, *Macromol. Chem. Phys.* **2002**, *203*, 889-899.

[3] J. Payne, M. D. Jones, *ChemSusChem* **2021**, *14*, 4041-4070.

[4] F. Liguori, C. Moreno-Marrodán, W. Oberhauser, E. Passaglia, P. Barbaro, *RSC Sustainability* **2023**, *1*, 1394-1403.

[5] M. Montrone, C. Cardellicchio, M. A. M. Capozzi, *Applied Sciences* **2025**, *15*.
